# Supplementary material for: Antifungal Polyacetylenic Deoxyglycosides Isolated from Endophytic Fungus Xylaria sp. VDL4 Associated with Vaccinium dunalianum
Source: J Fungi (Basel). 2025 Mar 8;11(3):209. doi: 10.3390/jof11030209 (PMC11943359; doi:10.3390/jof11030209)
Supplement: Supplementary file 1 [file jof-11-00209-s001.zip › jof-3444661-supplementary.pdf]

Supporting Information

Antifungal polyacetylenic deoxyglycosides isolated from endophytic fungus *Xylaria* sp. VDL4 associated with *Vaccinium dunalianum*

Jiao Yao [a,†], Sai Huang [a,†], Lingfeng He [a], Shengyun Wei [a], Wei Yang [a], Qiangxin Zhang [a], Weihua Wang [a,b], Xiaoqing Yang [a], Sida Xie [a], Yunxian Li [a], Ping Zhao [a,*], Guolei Zhu [a,*]

[a] *Key Laboratory of State Forestry Administration on Highly-Efficient Utilization of Forestry Biomass Resources in Southwest China, Southwest Forestry University, Kunming 650224, China*

[b] *Yunnan Key Laboratory of Gastrodia and Fungi Symbiotic Biology, Zhaotong University, Zhaotong 657000, China*

* Corresponding authors.

E-mail addresses: hypzhao2022@163.com (P. Zhao) and guoleizhu@163.com (G.L. Zhu).

† These authors made equal contribution to this work.

Contents of Supporting Information

Figure S1. ^{1}H NMR spectrum of **1** in methanol-d_4 1

Figure S2. ^{13}C NMR and DEPT spectra of **1** in methanol-d_4 1

Figure S3. HSQC spectrum of **1** in methanol-d_4 2

Figure S4. HMBC spectrum of **1** in methanol-d_4 2

Figure S5. ^{1}H-^{1}H COSY spectrum of **1** in methanol-d_4 3

Figure S6. NOESY spectrum of **1** in methanol-d_4 3

Figure S7. HRESIMS (+) spectrum of **1** 4

Figure S8. UV spectrum of **1** 5

Figure S9. The ^{1}H coupled HMQC spectrum for **1** (500 MHz) 5

Figure S10. IR spectrum of **1** 6

Figure S11. Optical rotation spectrum of **1** 7

Figure S13. ^{13}C NMR and DEPT spectrum of **2** in methanol-d_4 8

Figure S15. HMBC spectrum of **2** in methanol-d_4 9

Figure S16. ^{1}H-^{1}H COSY spectrum of **2** in methanol-d_4 9

Figure S17. NOESY spectrum of **2** in methanol-d_4 10

Figure S18. HRESIMS (+) spectrum of **2** 11

Figure S19. UV spectrum of **2** 12

Figure S22. ^{1}H NMR spectrum of **3** in methanol-d_4 14

Figure S23. ^{13}C NMR and DEPT spectrum of **3** in methanol-d_4 14

Figure S24. HSQC spectrum of **3** in methanol-d_4 15

Figure S25. HMBC spectrum of **3** in methanol-d_4 15

Figure S26. ^{1}H-^{1}H COSY spectrum of **3** in methanol-d_4 16

Figure S27. NOESY spectrum of **3** in methanol-d_4 16

Figure S28. HRESIMS (+) spectrum of **3** 17

Figure S29. UV spectrum of **3** 18

Figure S30. IR spectrum of **3** 19

Figure S31. Optical rotation spectrum of **3** 20

Figure S32. Fungal identification result. 20

Figure S33. Chemical structures of compounds **1–3** from *Xylaria sp.* 21

Figure S34. Key HMBC (red arrows) and ^{1}H- ^{1}H COSY correlations (blue bold lines) of **1–3** 21

Figure S35. Key ROESY correlations (blue double arrows) of **1–3**. 21

Figure S36. In α -glucosidase inhibitory test, concentration ranges and inhibition rates (I) of acarbose and Compounds 1-3.. 22

Figure S37. Fungal inhibition experiment of compounds **1-3**. 22

Figure S38. Summary of Experimental Data on *In vivo* Antifungal Activity Assay in Tomatoes 24

Figure S39. Summary of Experimental Data on *In vivo* Antifungal Activity Assay in Strawberry 25

Figure S1. ^{1}H NMR spectrum of **1** in methanol-d_4

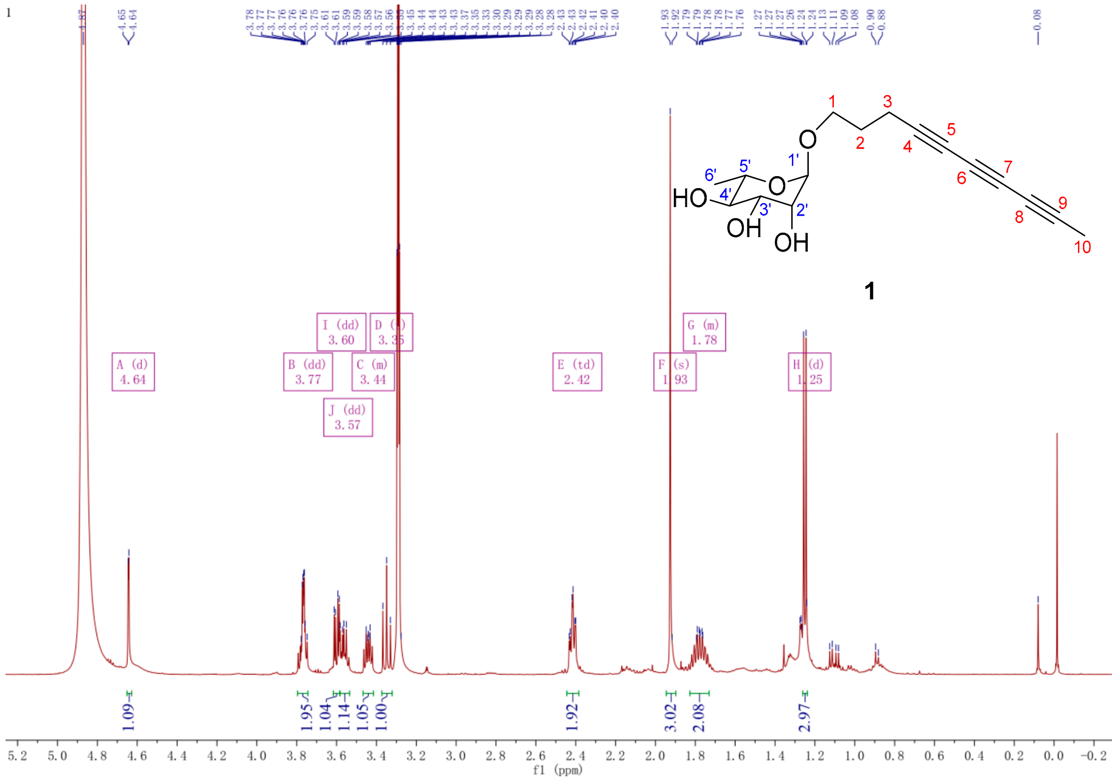

Figure S2. ^{13}C NMR and DEPT spectra of **1** in methanol-d_4

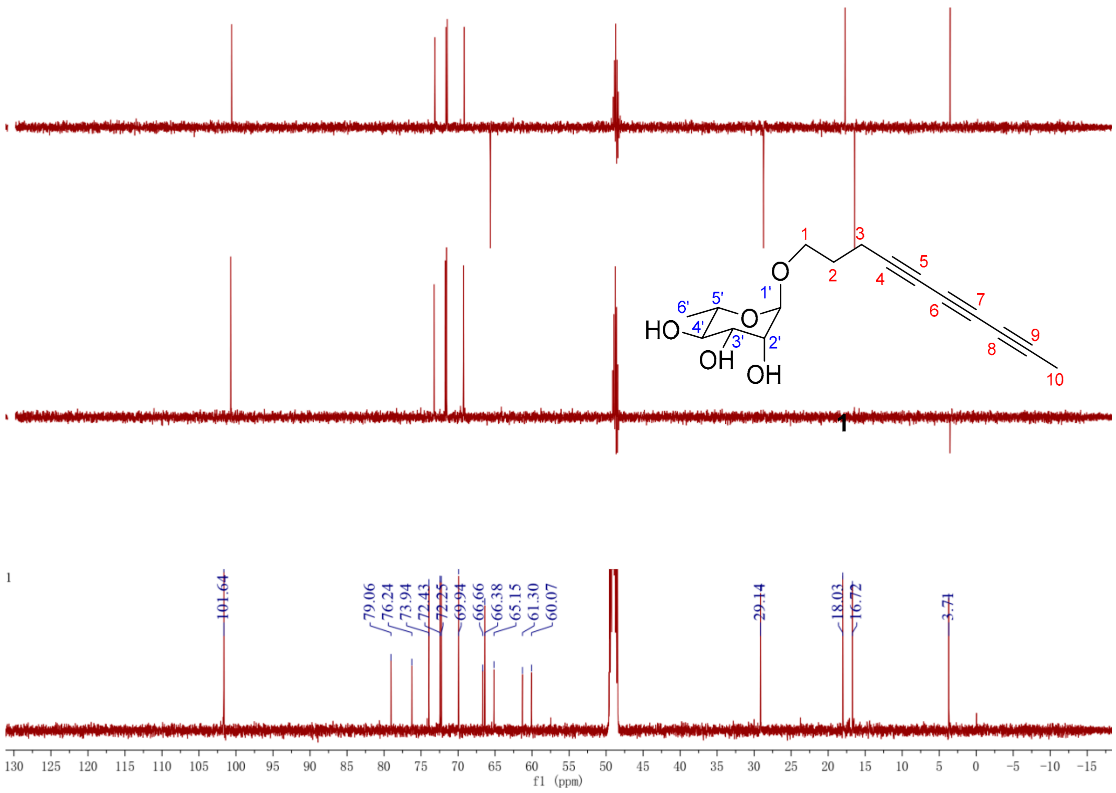

Figure S3. HSQC spectrum of **1** in methanol-d_4

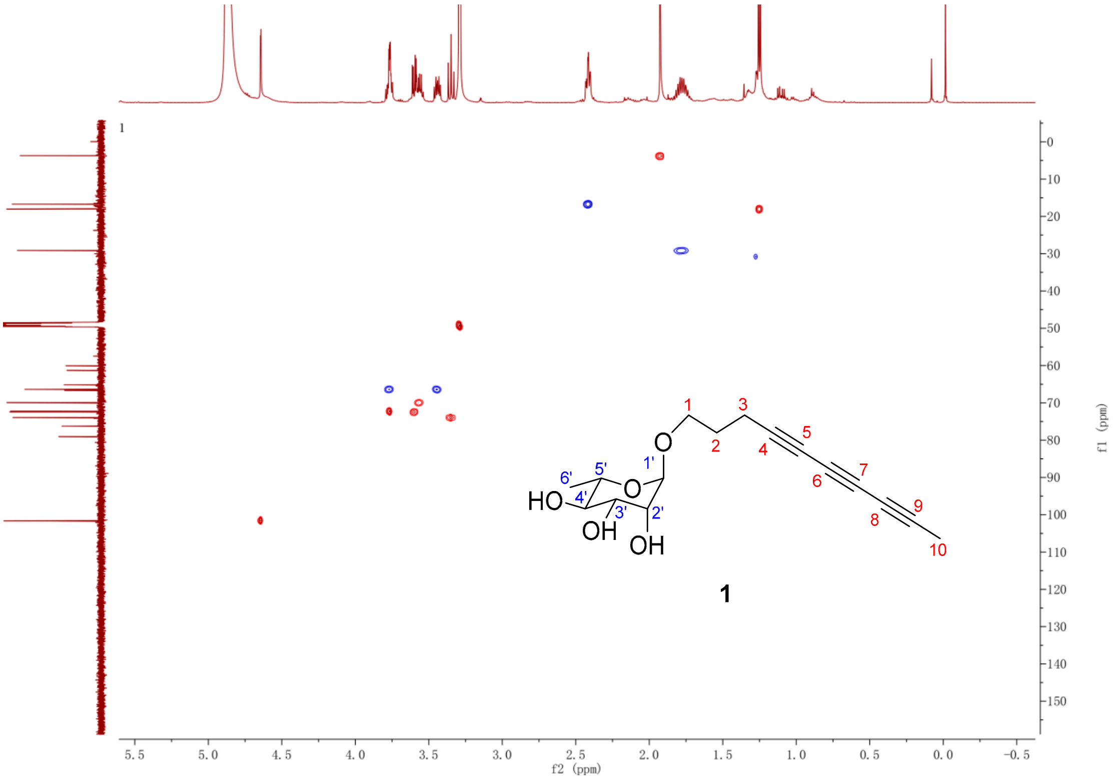

Figure S4. HMBC spectrum of **1** in methanol-d_4

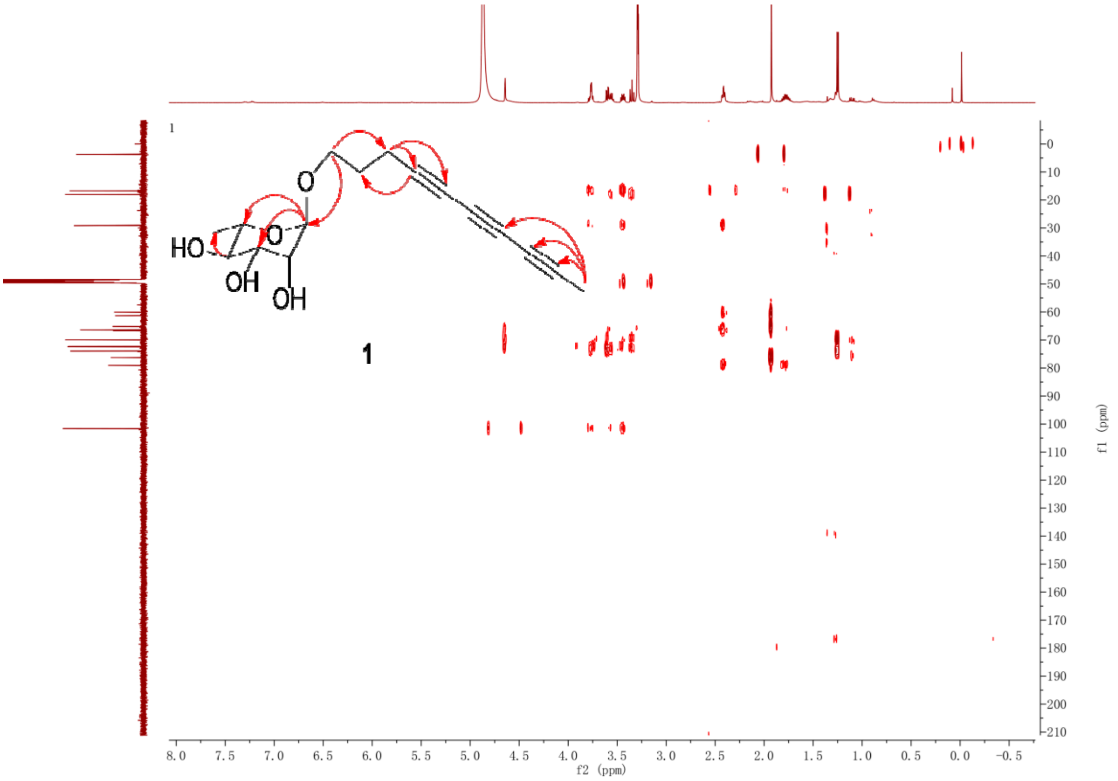

Figure S5. ^{1}H-^{1}H COSY spectrum of **1** in methanol-*d*$_4$

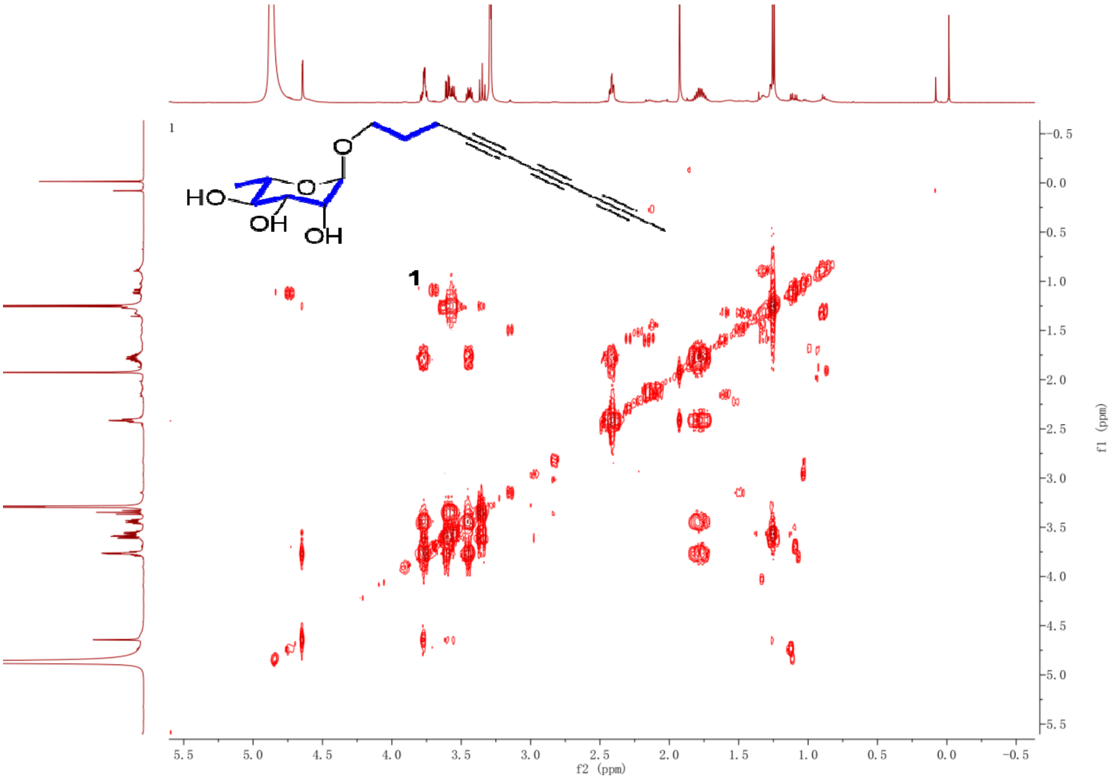

Figure S6. NOESY spectrum of **1** in methanol-*d*$_4$

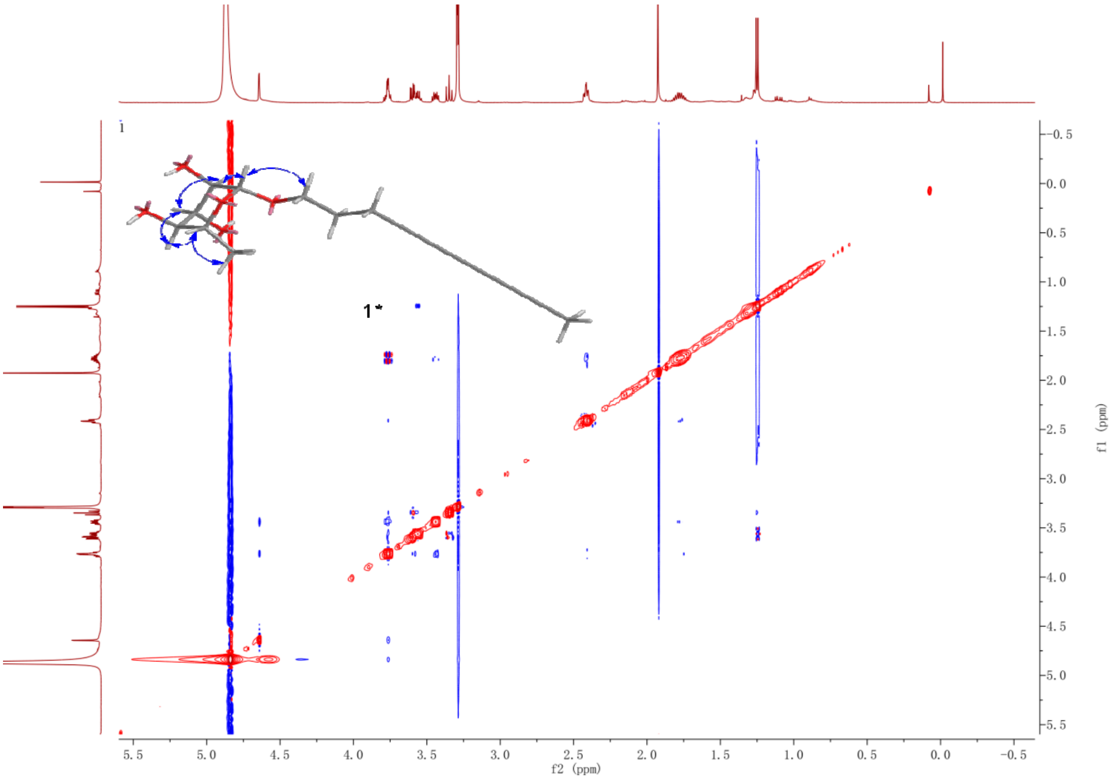

Figure S7. HRESIMS (+) spectrum of **1**

Qualitative Analysis Report

**Data Filename**	20240629ESIA4.d	**Sample Name**	YJ-8
**Sample Type**	Sample	**Position**	
**Instrument Name**	Agilent G6230 TOF MS	**User Name**	KIB
**Acq Method**	ESI.m	**Acquired Time**	6/29/2024 4:15:31 PM
**IRM Calibration Status**	Success	**DA Method**	ESI.m
**Comment**			

**Sample Group**		**Info.**
**Acquisition SW**	6200 series TOF/6500 series	
**Version**	Q-TOF B.05.01 (B5125.2)	

User Spectra

Fragmentor Voltage	Collision Energy	Ionization Mode
200	0	ESI

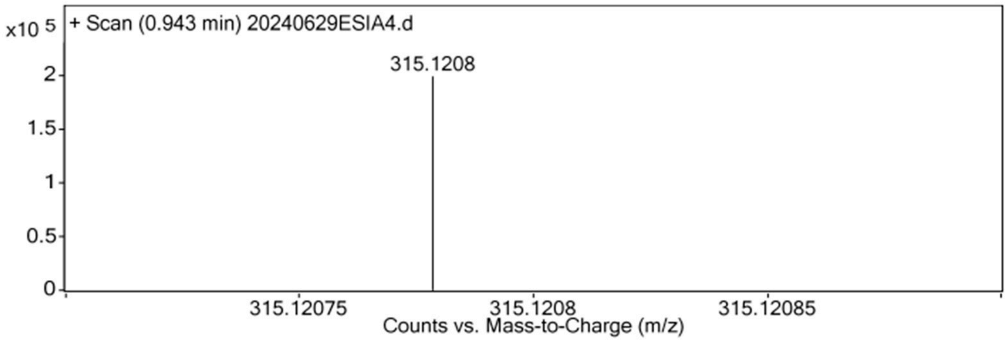

Peak List

m/z	z	Abund	Formula	Ion
121.0509	1	51210.75		
242.2839	1	33350.67		
309.1672	1	50641.71		
315.1208	1	198497.86	C16 H20 Na O5	M+
316.1234	1	27729.38	C16 H20 Na O5	M+
473.344	1	16370.17		
517.3693	1	15809.19		
607.2509	1	17648.22		
922.0098	1	98198.77		
923.0116	1	15120.04		

Formula Calculator Element Limits

Element	Min	Max
C	0	200
H	0	400
O	0	15
Na	1	1

Formula Calculator Results

Formula	CalculatedMass	Mz	Diff.(mDa)	Diff. (ppm)	DBE
C16 H20 Na O5	315.1208	315.1208	0.0	0.1	6.5

--- End Of Report ---

Figure S8. UV spectrum of **1**

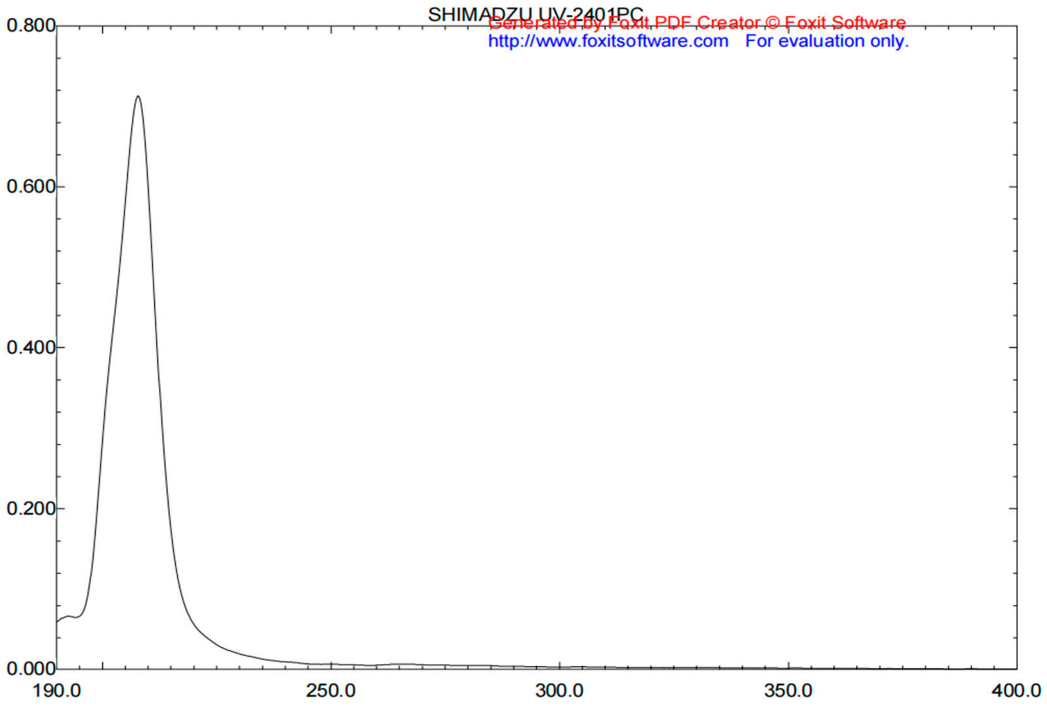

Figure S9. The ^{1}H coupled HMQC spectrum for **1** (500 MHz)

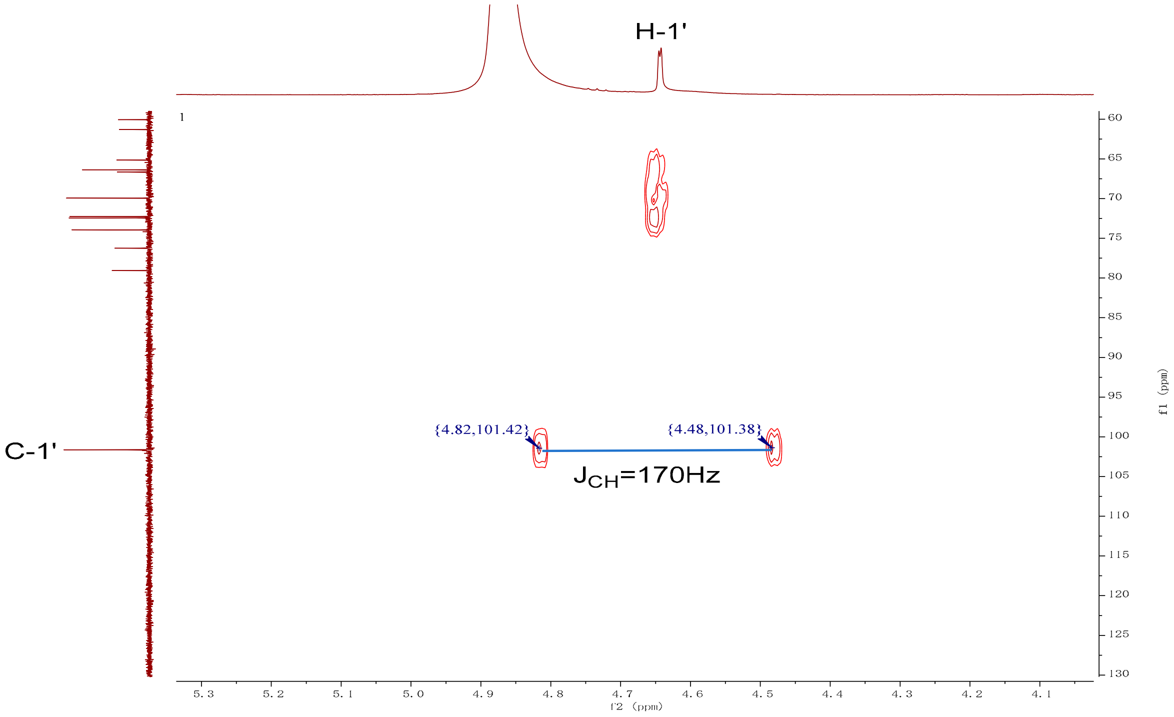

Figure S10. IR spectrum of **1**

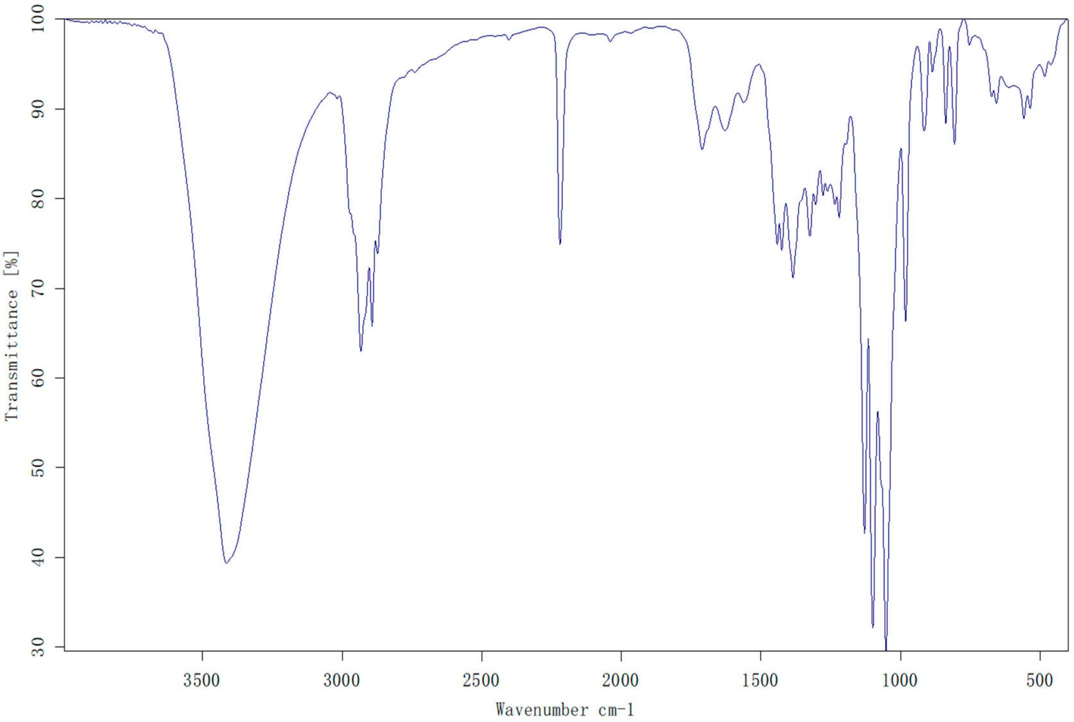

Sample Name: YJ-8
Sample Form: KBr
Path of File: E:\data
Date of Measurement: 2024/9/13

Resolution: 4
Aperture Setting: 6 mm
Number of Background Scans: 16
Number of Sample Scans: 16

Beamsplitter Setting: KBr
Source Setting: MIR
Instrument Type: BRUKER VERTEX 70
Soft Version: OPUS8.1

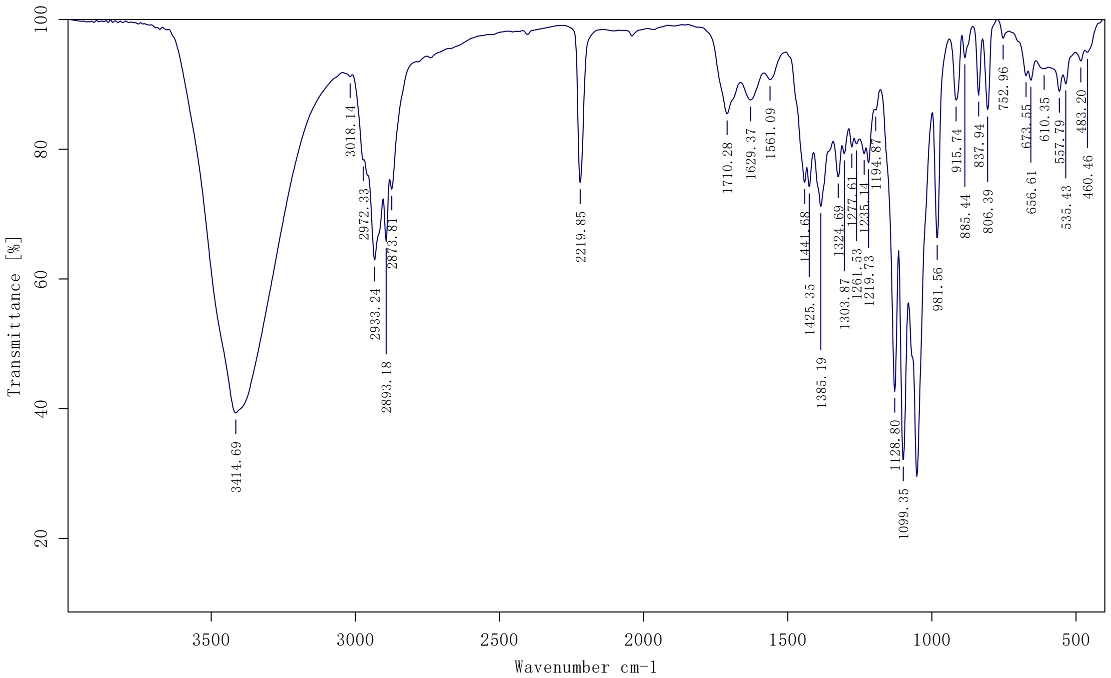

Sample Name: YJ-8
Sample Form: KBr
Path of File: E:\data
Date of Measurement: 2024/9/13

Resolution: 4
Aperture Setting: 6 mm
Number of Background Scans: 16
Number of Sample Scans: 16

Beamsplitter Setting: KBr
Source Setting: MIR
Instrument Type: BRUKER VERTEX 70
Soft Version: OPUS8.1

Figure S11. Optical rotation spectrum of **1**

Rudolph Research Analytical

This sample was measured on an Autopol VI, Serial #91058
Manufactured by Rudolph Research Analytical, Hackettstown, NJ, USA.

Measurement Date : Saturday, 13-JUL-2024

Set Temperature : 25.0

Time Delay : Disabled

Delay between Measurement : Disabled

n	Average	Std.Dev.	% RSD	Maximum	Minimum
5	-107.82	0.50	-0.46	-107.27	-108.18

S.No	Sample ID	Time	Result	Scale	OR °Arc	WLG.nm	Lg.mm	Conc.g/100ml	Temp.
1	YJ-8	10:59:49 AM	-108.18	SR	-0.119	589	100.00	0.110	25.0
2	YJ-8	10:59:55 AM	-108.18	SR	-0.119	589	100.00	0.110	25.0
3	YJ-8	11:00:02 AM	-108.18	SR	-0.119	589	100.00	0.110	25.0
4	YJ-8	11:00:08 AM	-107.27	SR	-0.118	589	100.00	0.110	25.0
5	YJ-8	11:00:14 AM	-107.27	SR	-0.118	589	100.00	0.110	25.0

Figure S12. ^{1}H NMR spectrum of **2** in methanol-d_4

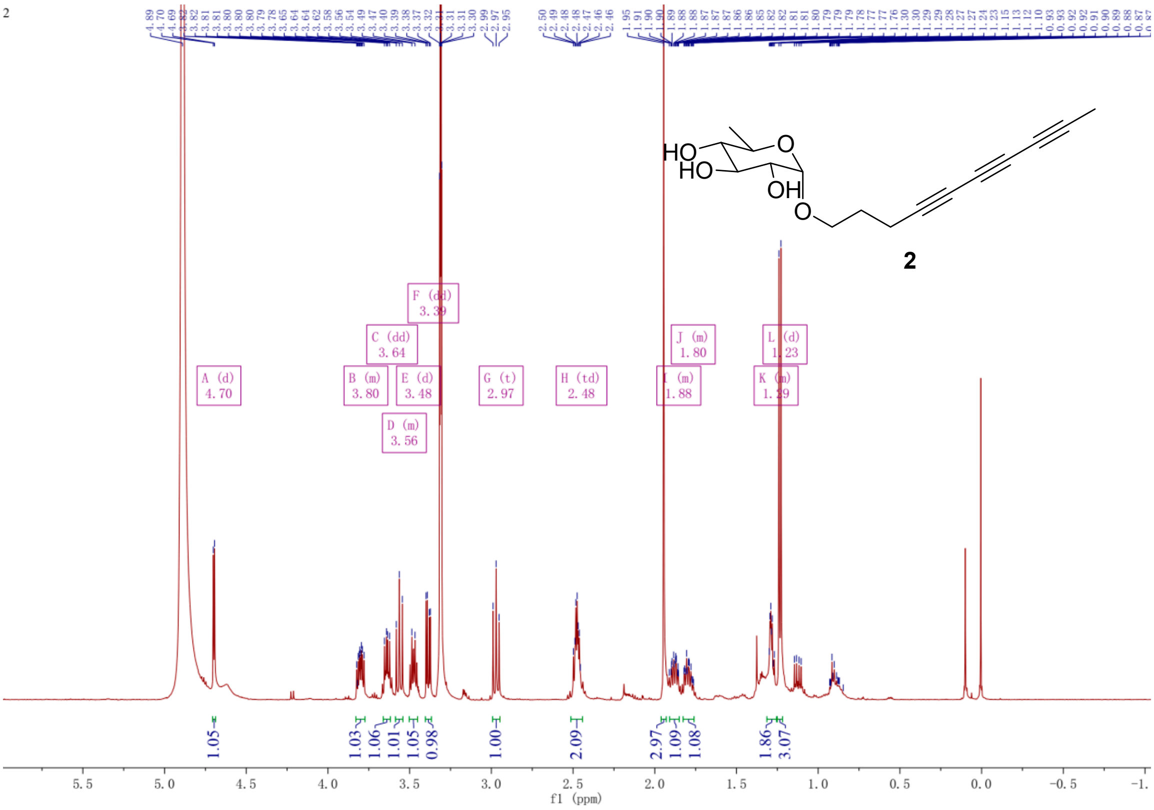

Figure S13. ^{13}C NMR and DEPT spectrum of **2** in methanol-d_4

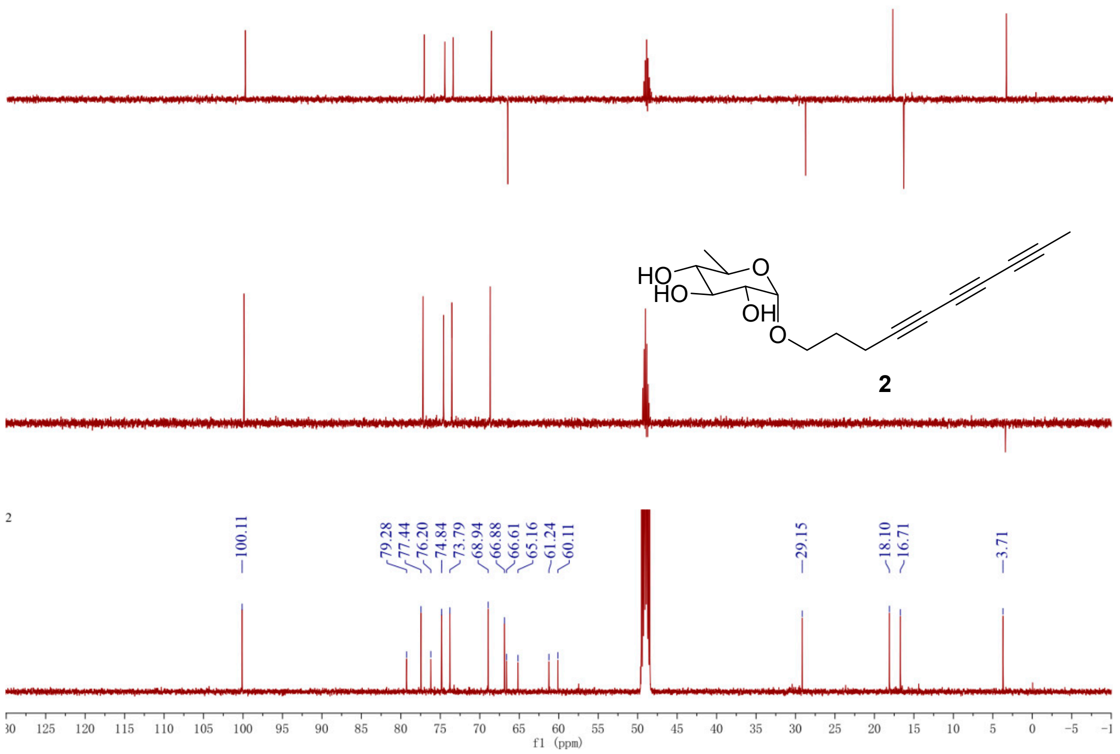

Figure S14. HSQC spectrum of **2** in methanol-d_4

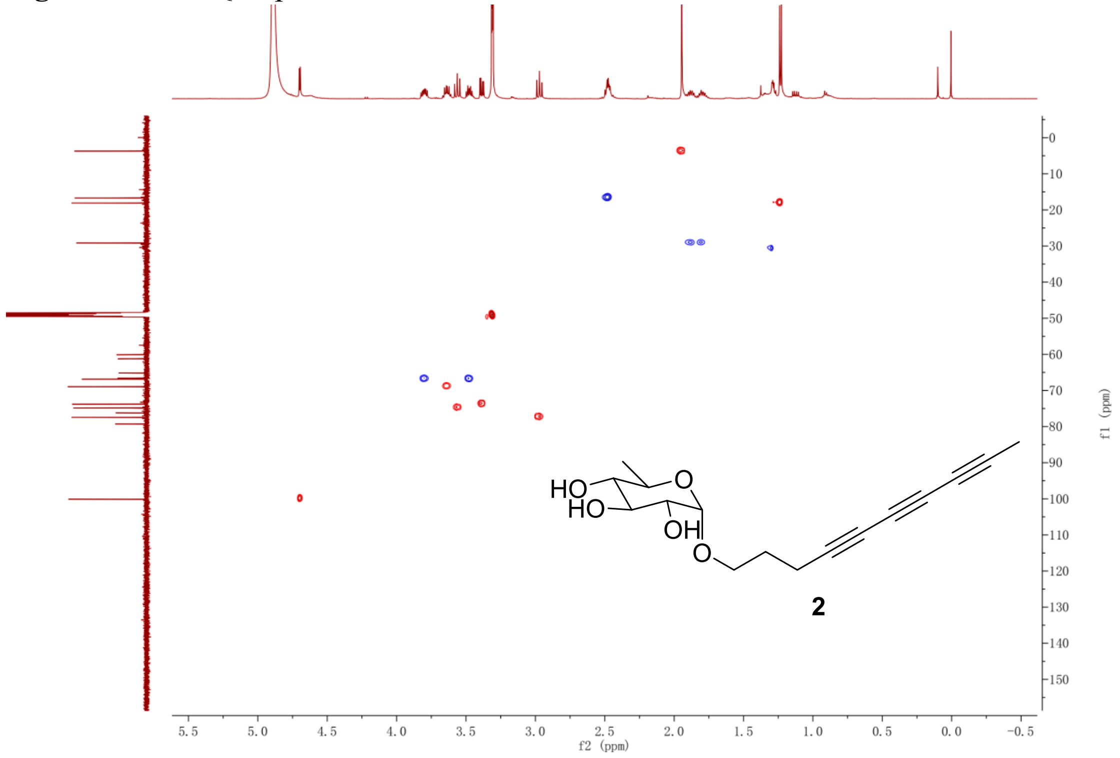

Figure S15. HMBC spectrum of **2** in methanol-d_4

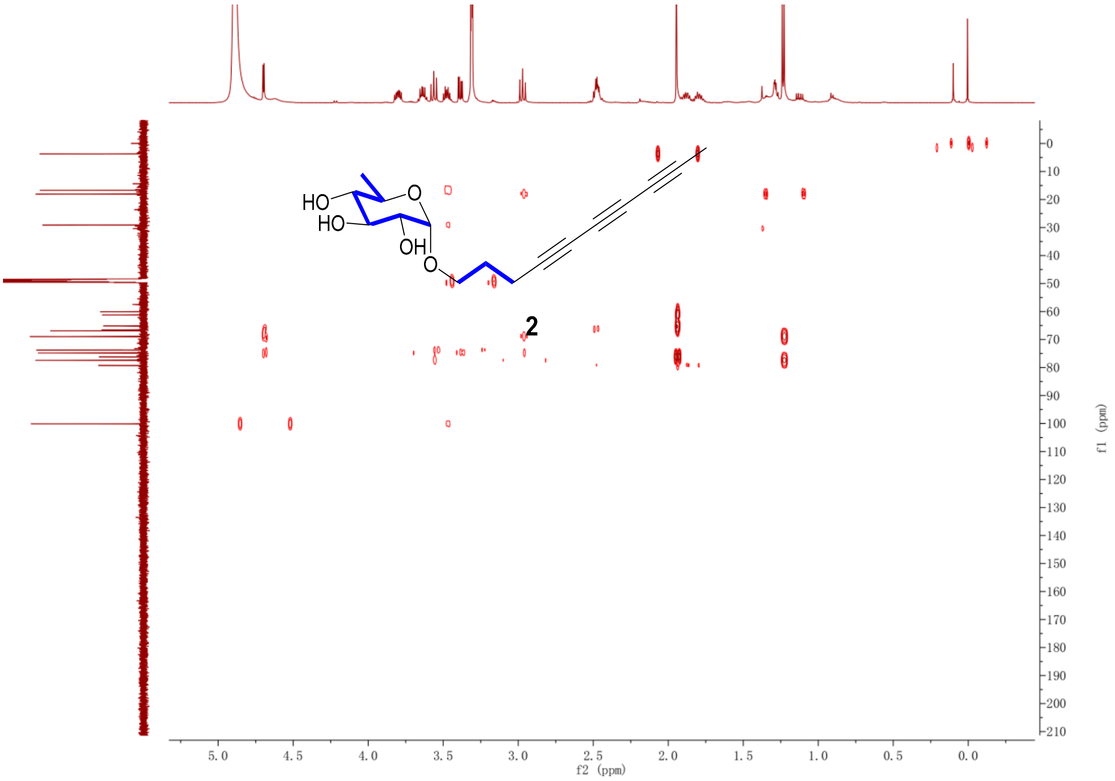

Figure S16. ^{1}H-^{1}H COSY spectrum of **2** in methanol-d_4

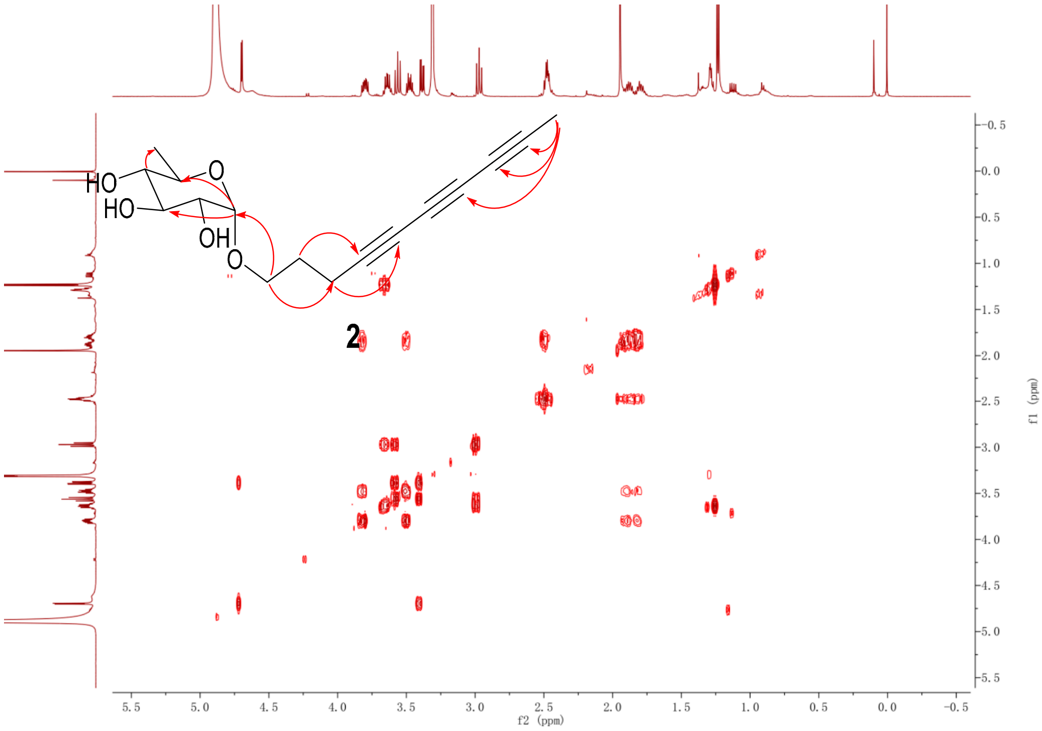

Figure S17. NOESY spectrum of **2** in methanol-*d₄*

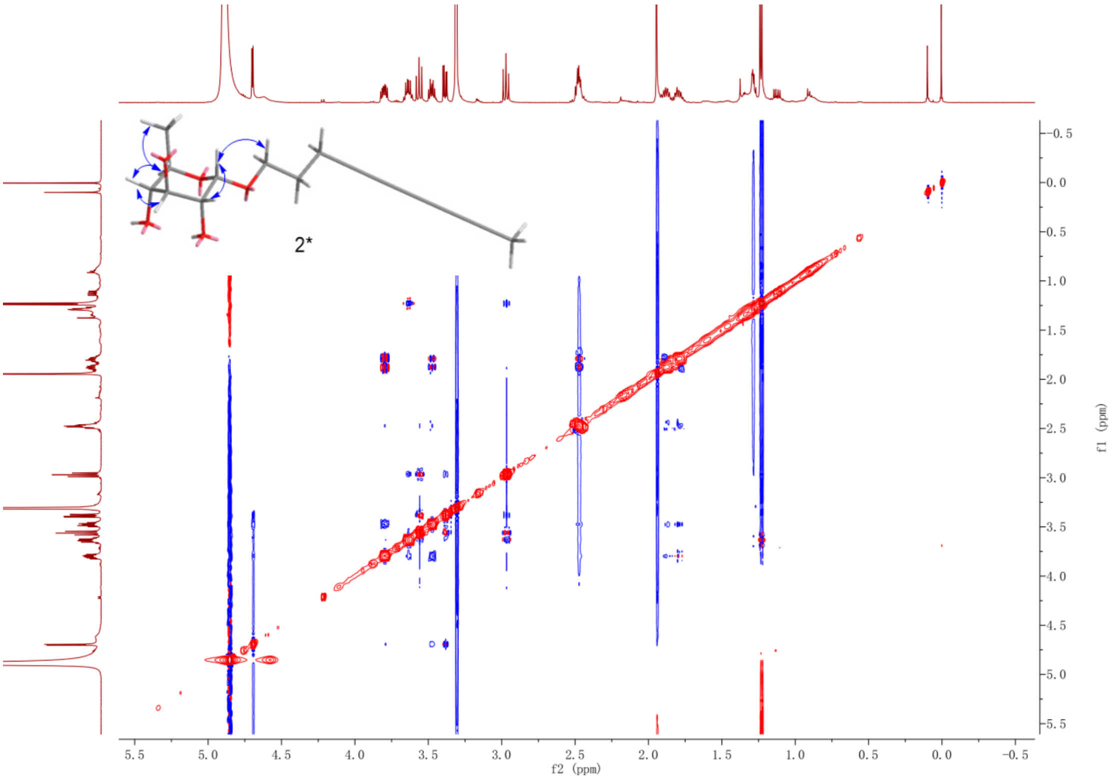

Figure S18. HRESIMS (+) spectrum of **2**

Qualitative Analysis Report

**Data Filename**	20240629ESIA3.d	**Sample Name**	YJ-7
**Sample Type**	Sample	**Position**	
**Instrument Name**	Agilent G6230 TOF MS	**User Name**	KIB
**Acq Method**	ESI.m	**Acquired Time**	6/29/2024 4:13:35 PM
**IRM Calibration Status**	Success	**DA Method**	ESI.m
**Comment**			

**Sample Group**		**Info.**
**Acquisition SW Version**	6200 series TOF/6500 series Q-TOF B.05.01 (B5125.2)	

User Spectra

Fragmentor Voltage	Collision Energy	Ionization Mode
200	0	ESI

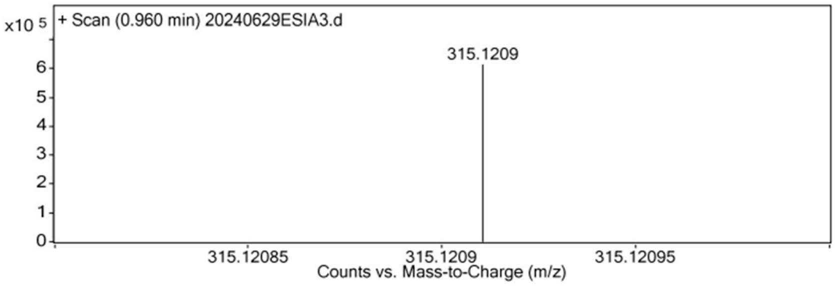

Peak List

m/z	z	Abund	Formula	Ion
121.0509	1	87916.83		
293.1745	1	15616.02		
309.167	1	80941.98		
315.1209	1	612800.88	C16 H20 Na O5	M+
316.1235	1	93078.6	C16 H20 Na O5	M+
364.2533	1	20952.58		
437.1939	1	26206.1		
607.2499	1	37459.79		
922.0098	1	150990.05		
923.0114	1	22405.07		

Formula Calculator Element Limits

Element	Min	Max
C	0	200
H	0	400
O	0	15
Na	1	1

Formula Calculator Results

Formula	CalculatedMass	Mz	Diff.(mDa)	Diff. (ppm)	DBE
C16 H20 Na O5	315.1208	315.1209	-0.1	0.2	6.5

--- End Of Report ---

Figure S19. UV spectrum of **2**

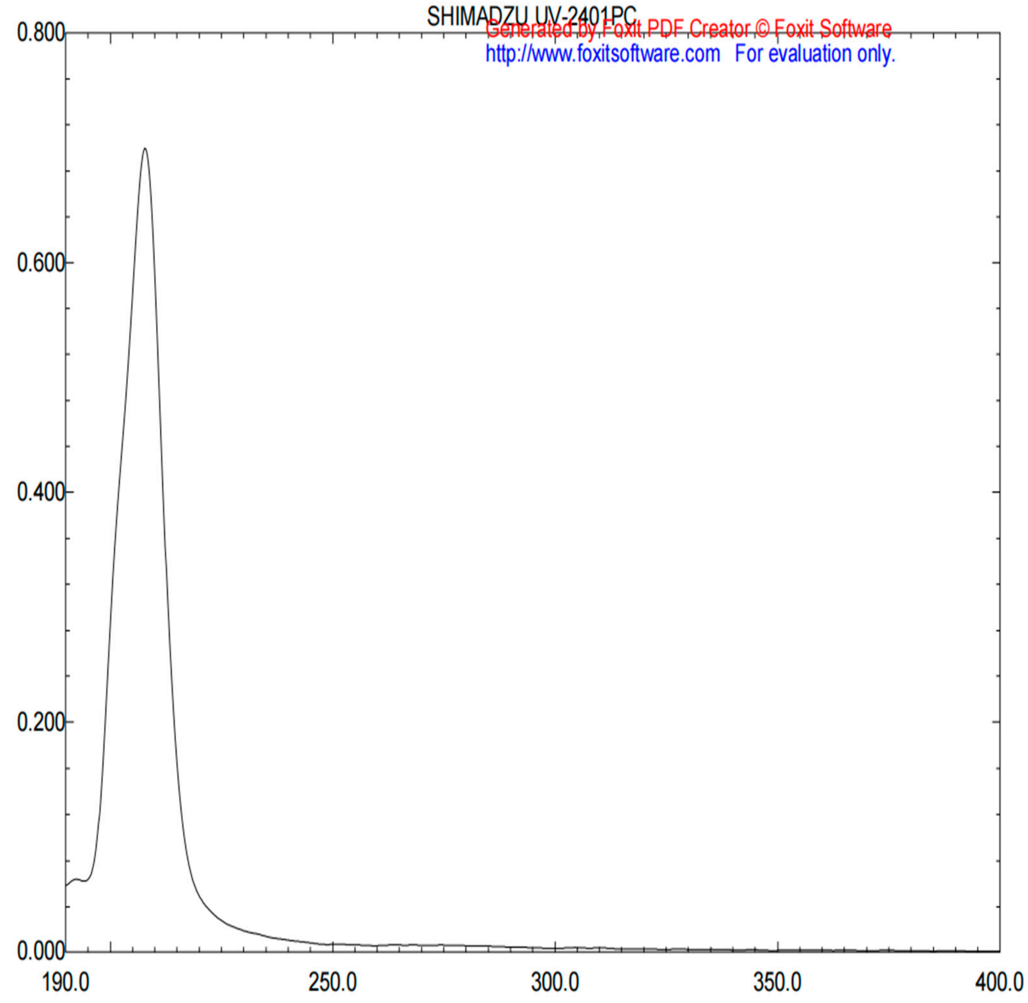

Generated by Foxit PDF Creator © Foxit Software
http://www.foxitsoftware.com For evaluation only.

Figure S20. IR spectrum of **2**

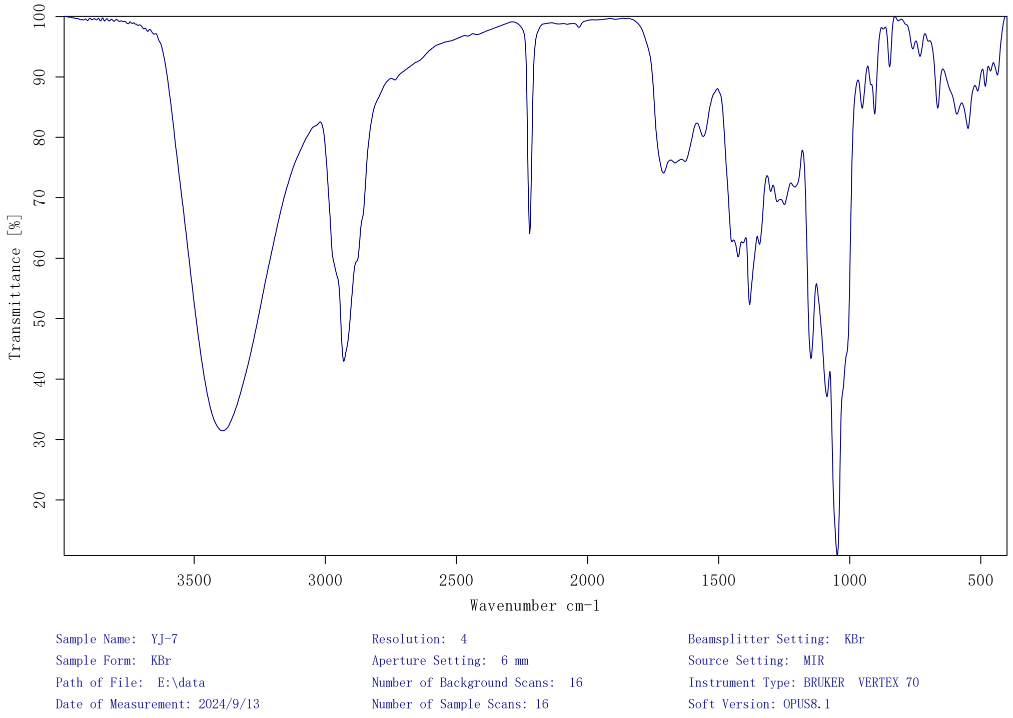

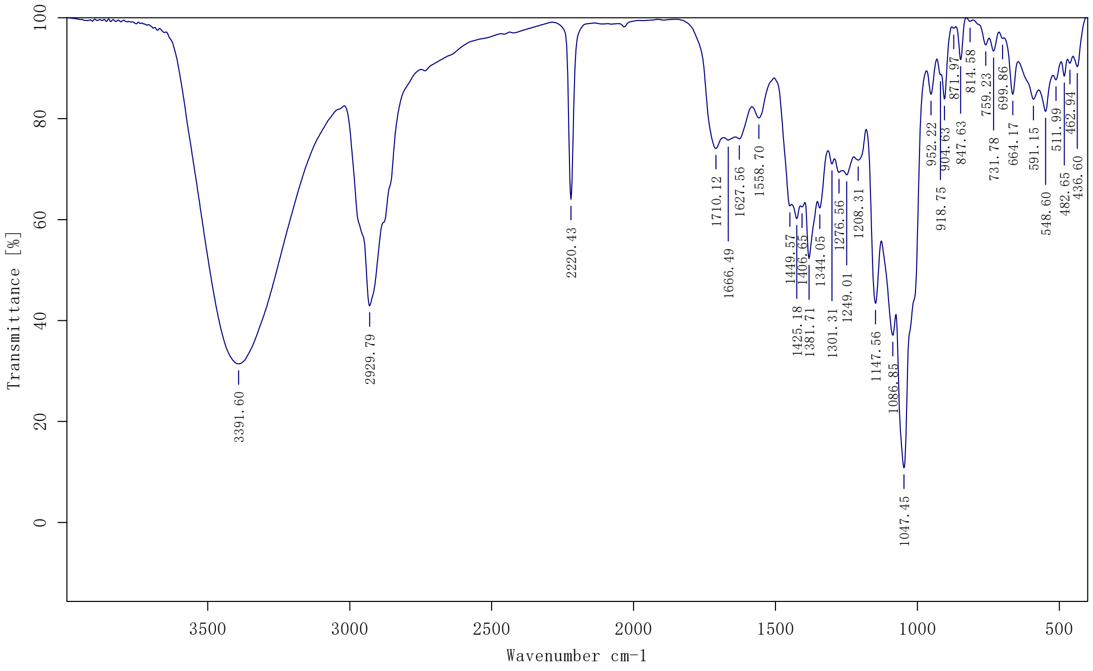

Sample Name: YJ-7
Sample Form: KBr
Path of File: E:\data
Date of Measurement: 2024/9/13

Resolution: 4
Aperture Setting: 6 mm
Number of Background Scans: 16
Number of Sample Scans: 16

Beamsplitter Setting: KBr
Source Setting: MIR
Instrument Type: BRUKER VERTEX 70
Soft Version: OPUS8.1

Figure S21. Optical rotation spectrum of **2**

Rudolph Research Analytical

This sample was measured on an Autopol VI, Serial #91058
Manufactured by Rudolph Research Analytical, Hackettstown, NJ, USA.

Measurement Date : Saturday, 13-JUL-2024

Set Temperature : 25.0

Time Delay : Disabled

Delay between Measurement : Disabled

n	Average	Std.Dev.	% RSD	Maximum	Minimum
5	-146.67	0.00	0.00	-146.67	-146.67

S.No	Sample ID	Time	Result	Scale	OR °Arc	WLG.nm	Lg.mm	Conc.g/100ml	Temp.
1	YJ-7	10:55:11 AM	-146.67	SR	-0.176	589	100.00	0.120	25.0
2	YJ-7	10:55:17 AM	-146.67	SR	-0.176	589	100.00	0.120	25.0
3	YJ-7	10:55:24 AM	-146.67	SR	-0.176	589	100.00	0.120	25.0
4	YJ-7	10:55:30 AM	-146.67	SR	-0.176	589	100.00	0.120	25.0
5	YJ-7	10:55:36 AM	-146.67	SR	-0.176	589	100.00	0.120	25.0

Figure S22. ^{1}H NMR spectrum of **3** in methanol-d_4

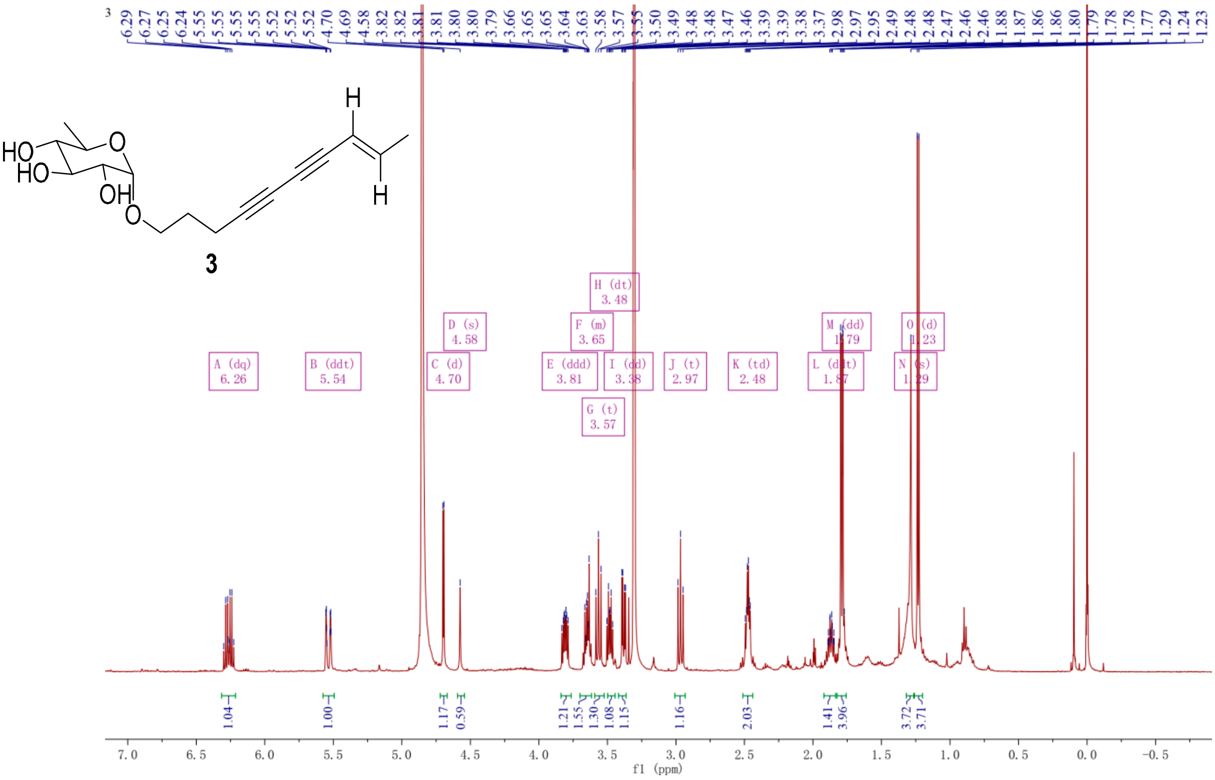

Figure S23. ^{13}C NMR and DEPT spectrum of **3** in methanol-d_4

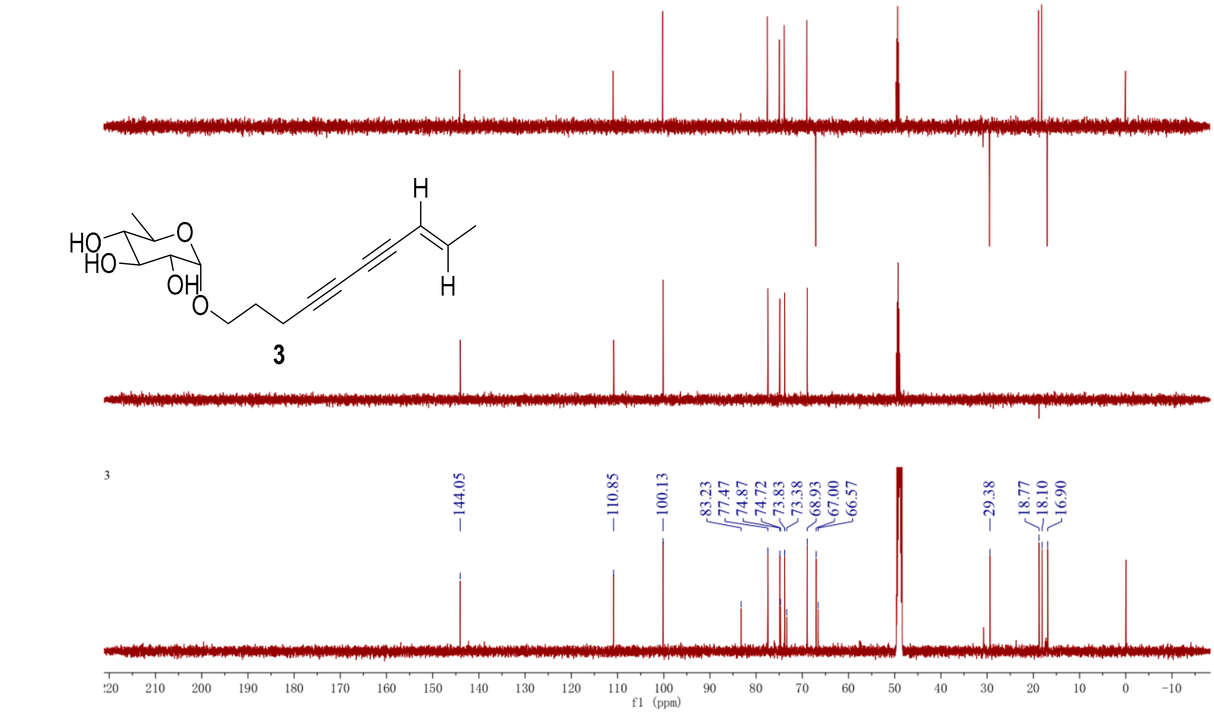

Figure S24. HSQC spectrum of **3** in methanol-*d*4

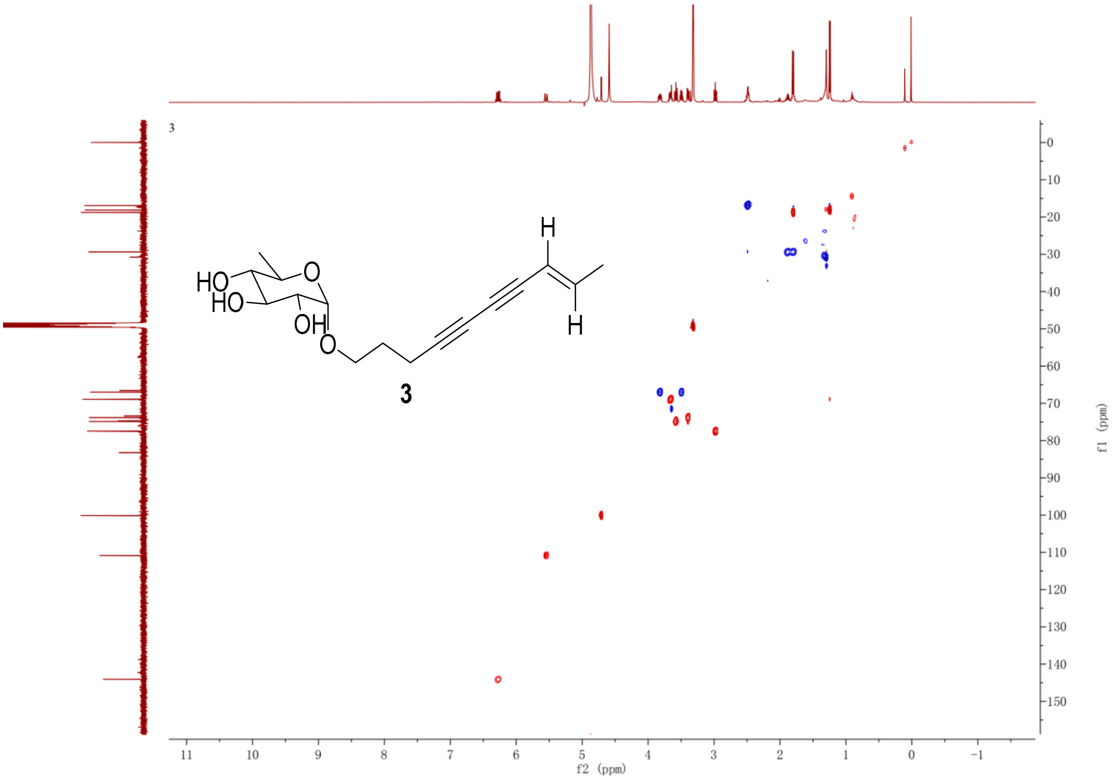

Figure S25. HMBC spectrum of **3** in methanol-*d*4

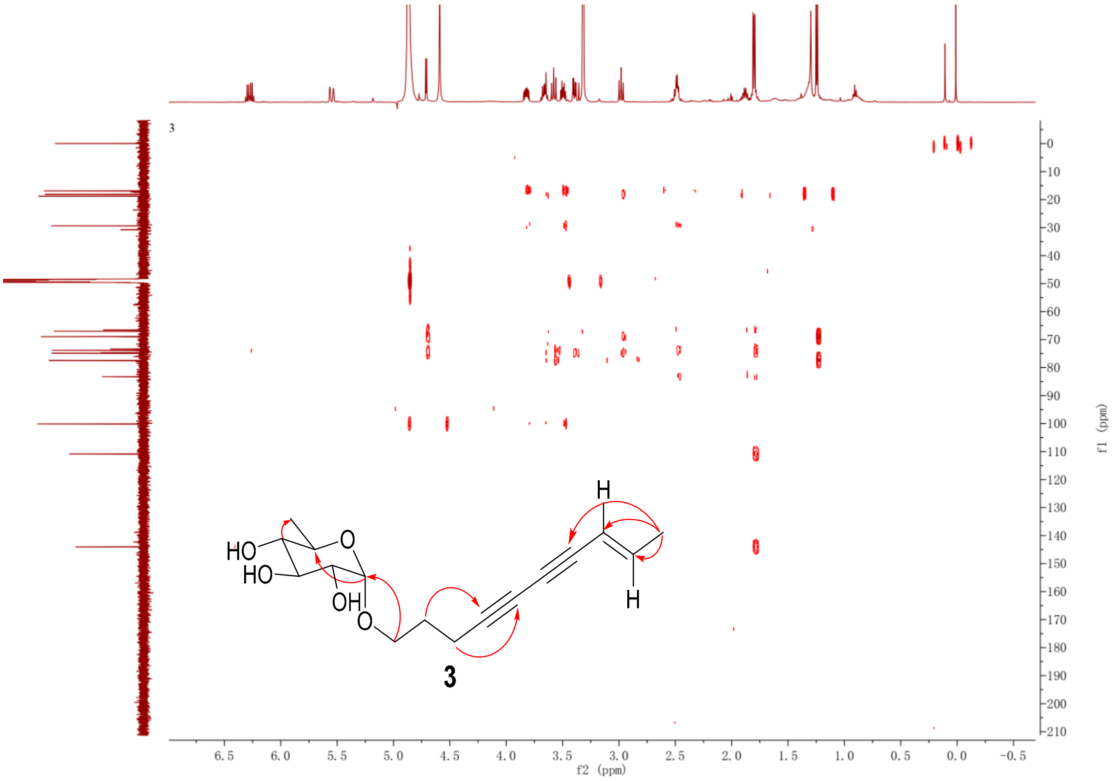

Figure S26. ^1H-^1H COSY spectrum of **3** in methanol-d_4

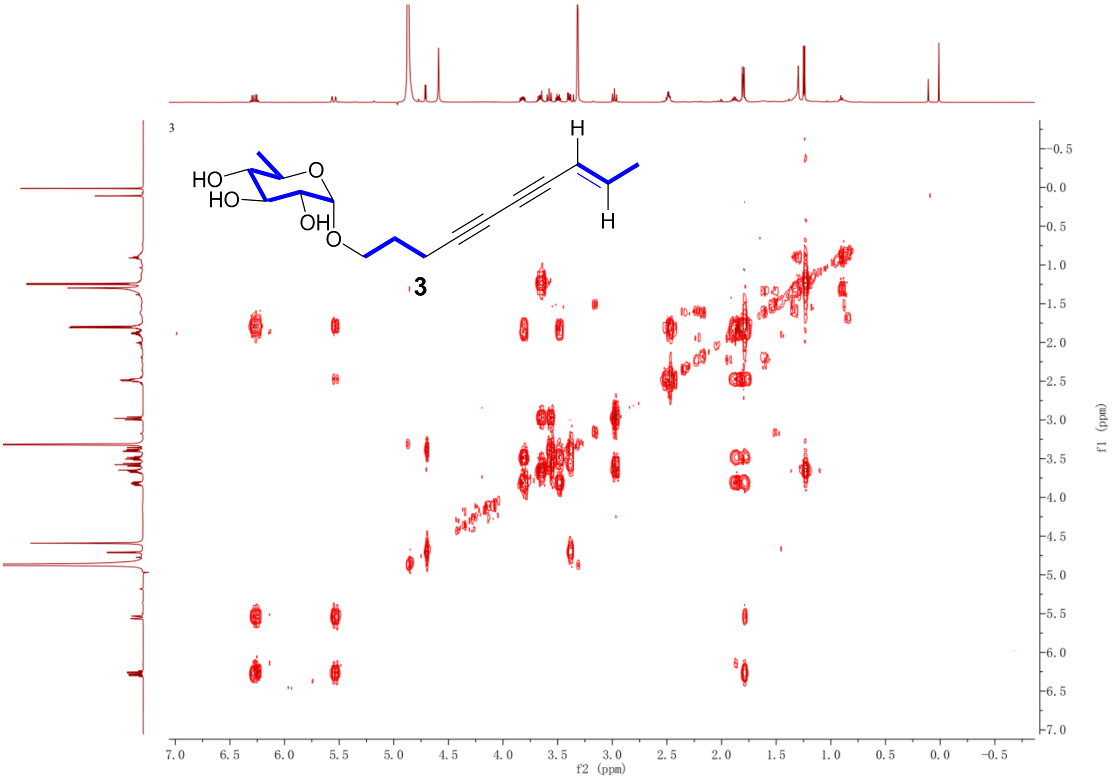

Figure S27. NOESY spectrum of **3** in methanol-d_4

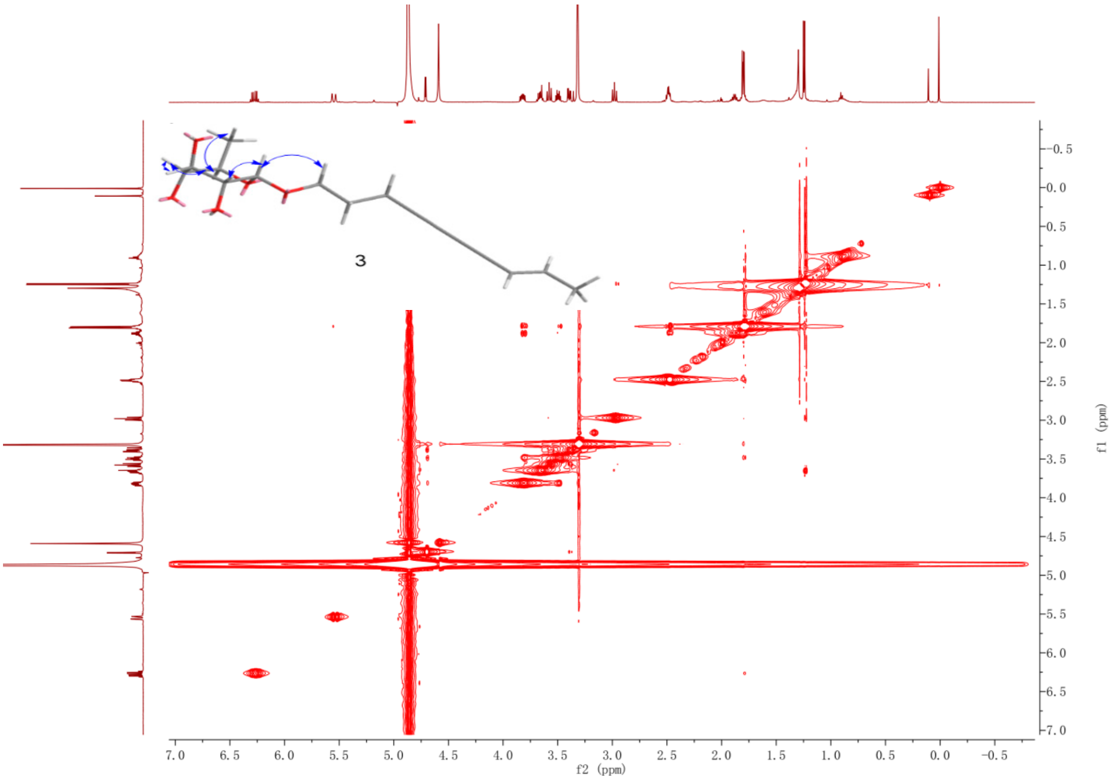

Figure S28. HRESIMS (+) spectrum of **3**

Qualitative Analysis Report

**Data Filename**	20240906ESIA26.d	**Sample Name**	YJ-90-1
**Sample Type**	Sample	**Position**	Vial 21
**Instrument Name**	Agilent G6230 TOF MS	**User Name**	
**Acq Method**	ESI.m	**Acquired Time**	9/6/2024 2:57:57 PM
**IRM Calibration Status**	Success	**DA Method**	ESI.m
**Comment**			

Sample Group **Info.**
Acquisition SW Version 6200 series TOF/6500 series Q-TOF B.05.01 (B5125.2)

User Spectra

Fragmentor Voltage	Collision Energy	Ionization Mode
200	0	ESI

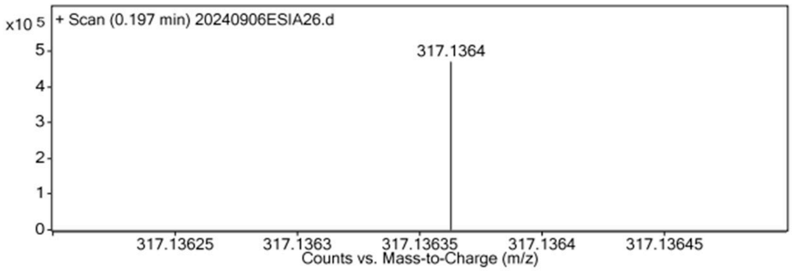

Peak List

m/z	z	Abund	Formula	Ion
102.1279		26342.88		
121.0509	1	44070.04		
142.1585	1	71173.46		
186.2209	1	52918.63		
242.2845	1	1093762.13		
243.2874	1	180806.39		
317.1364	1	470428.5	C16 H22 Na O5	M+
318.1389	1	68909.35	C16 H22 Na O5	M+
437.1934	1	64104.41		
922.0098	1	98646.53		

Formula Calculator Element Limits

Element	Min	Max
C	0	200
H	0	400
O	0	10
Na	1	1

Formula Calculator Results

Formula	CalculatedMass	Mz	Diff.(mDa)	Diff. (ppm)	DBE
C16 H22 Na O5	317.1365	317.1364	0.1	0.3	5.5

--- End Of Report ---

Figure S29. UV spectrum of **3**

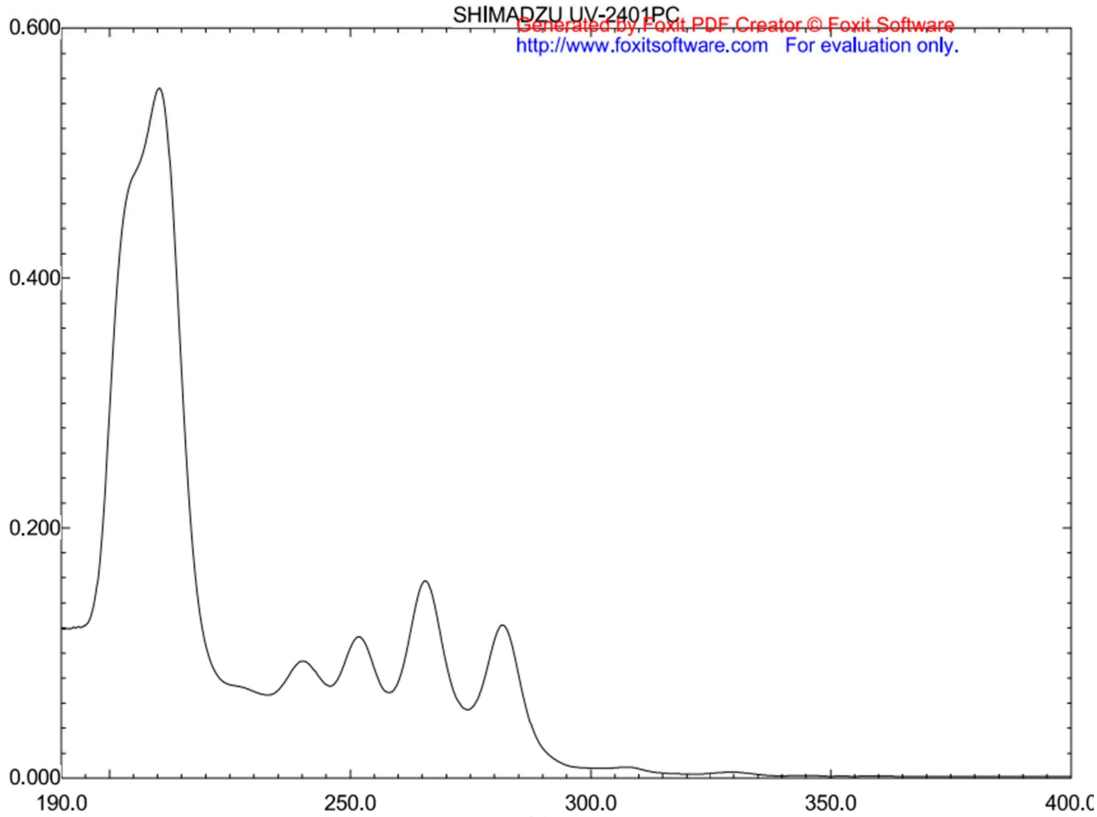

Generated by Foxit PDF Creator © Foxit Software
http://www.foxitsoftware.com For evaluation only.

Figure S30. IR spectrum of **3**

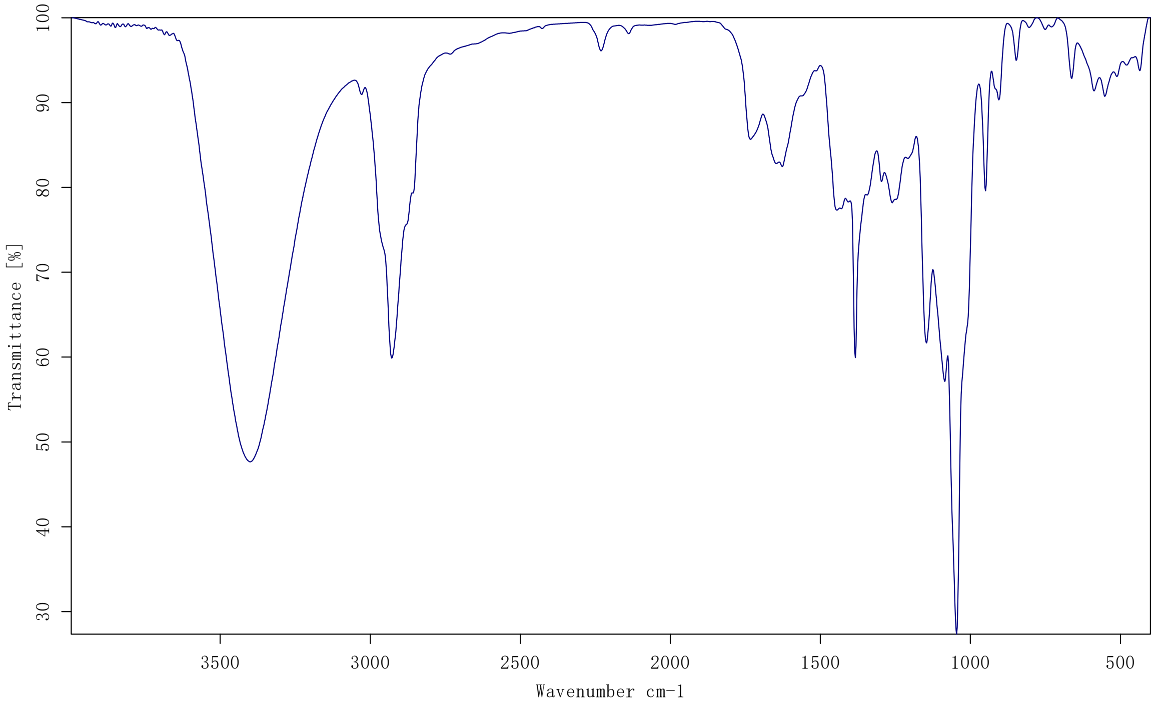

Sample Name: YJ-90
Sample Form: KBr
Path of File: E:\data
Date of Measurement: 2024/9/13

Resolution: 4
Aperture Setting: 6 mm
Number of Background Scans: 16
Number of Sample Scans: 16

Beamsplitter Setting: KBr
Source Setting: MIR
Instrument Type: BRUKER VERTEX 70
Soft Version: OPUS8.1

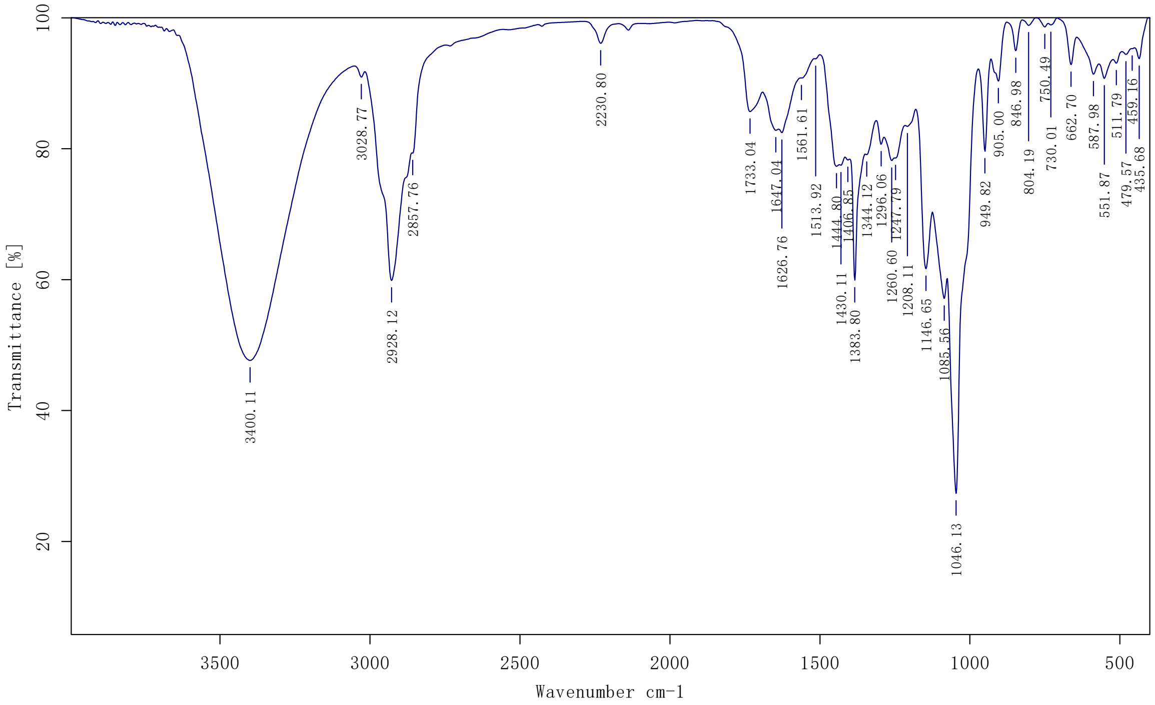

Sample Name: YJ-90
Sample Form: KBr
Path of File: E:\data
Date of Measurement: 2024/9/13

Resolution: 4
Aperture Setting: 6 mm
Number of Background Scans: 16
Number of Sample Scans: 16

Beamsplitter Setting: KBr
Source Setting: MIR
Instrument Type: BRUKER VERTEX 70
Soft Version: OPUS8.1

Figure S31. Optical rotation spectrum of **3**

Rudolph Research Analytical

This sample was measured on an Autopol VI, Serial #91058
Manufactured by Rudolph Research Analytical, Hackettstown, NJ, USA.

Measurement Date : Friday, 06-SEP-2024

Set Temperature : 25.0

Time Delay : Disabled

Delay between Measurement : Disabled

n	Average	Std.Dev.	% RSD	Maximum	Minimum
5	-109.40	0.55	-0.50	-109.00	-110.00

S.No	Sample ID	Time	Result	Scale	OR °Arc	WLG.nm	Lg.mm	Conc.g/100ml	Temp.
1	YJ-90	07:48:53 PM	-110.00	SR	-0.110	589	100.00	0.100	25.1
2	YJ-90	07:48:59 PM	-109.00	SR	-0.109	589	100.00	0.100	25.1
3	YJ-90	07:49:05 PM	-109.00	SR	-0.109	589	100.00	0.100	25.0
4	YJ-90	07:49:11 PM	-110.00	SR	-0.110	589	100.00	0.100	25.0
5	YJ-90	07:49:17 PM	-109.00	SR	-0.109	589	100.00	0.100	25.0

Figure S32. Fungal identification result.

Description	Scientific Name	Max Score	Total Score	Query Cover	E value	Per. Ident	Acc. Len	Accession
Xylaria sp. strain KUNCC22-10746 small subunit ribosomal RNA gene, partial sequence; internal transcribed spa...	Xylaria sp.	1020	1020	99%	0.0	100.00%	570	ON520749.1
Xylaria sp. strain KUNCC22-10745 small subunit ribosomal RNA gene, partial sequence; internal transcribed spa...	Xylaria sp.	1020	1020	99%	0.0	100.00%	573	ON520748.1
Fungal sp. isolate NBRI_PD1_Xylaria_sp. small subunit ribosomal RNA gene, partial sequence; internal transcrib...	fungal sp.	1007	1007	98%	0.0	100.00%	593	ON876166.1
Xylaria sp. strain LTL43 small subunit ribosomal RNA gene, partial sequence; internal transcribed spacer 1, 5.8S ...	Xylaria sp.	992	992	99%	0.0	99.09%	590	MF663544.1
Xylaria sp. 1 JHY-2020a isolate KoLRI49039 internal transcribed spacer 1, partial sequence; 5.8S ribosomal RNA...	Xylaria sp. 1 JHY...	987	987	98%	0.0	99.45%	556	MT586903.1
Xylaria sp. 5156 18S ribosomal RNA gene, partial sequence; internal transcribed spacer 1, 5.8S ribosomal RNA g...	Xylaria sp. 5156	961	961	95%	0.0	99.62%	567	JX868518.1
Xylariaceae sp. isolate A465 small subunit ribosomal RNA gene, partial sequence; internal transcribed spacer 1 a...	Xylariaceae sp.	955	955	94%	0.0	99.81%	544	MK247682.1
Xylaria sp. isolate GG3F35 internal transcribed spacer 1, partial sequence; 5.8S ribosomal RNA gene and interna...	Xylaria sp.	955	955	94%	0.0	99.81%	523	KY419565.1
Fungal sp. P6N12a internal transcribed spacer 1, partial sequence; 5.8S ribosomal RNA gene and internal transc...	fungal sp. P6N12a	955	955	95%	0.0	99.43%	530	KF735016.1
Xylaria sp. strain 1184 internal transcribed spacer 1, partial sequence; 5.8S ribosomal RNA gene and internal tra...	Xylaria sp.	955	955	97%	0.0	98.70%	558	MZ400557.1
Xylaria sp. 5165 internal transcribed spacer 1, partial sequence; 5.8S ribosomal RNA gene and internal transcrib...	Xylaria sp. 5165	955	955	95%	0.0	99.25%	567	JQ862667.1
Xylaria sp. isolate Otu0088 small subunit ribosomal RNA gene, partial sequence; internal transcribed spacer 1 an...	Xylaria sp.	955	955	94%	0.0	99.81%	544	MT908464.1

Figure S33. Chemical structures of compounds **1–3** from *Xylaria sp.*

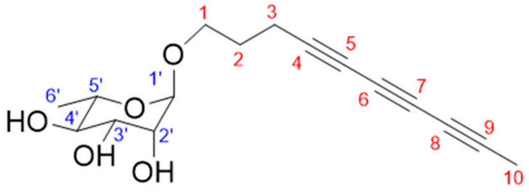

1

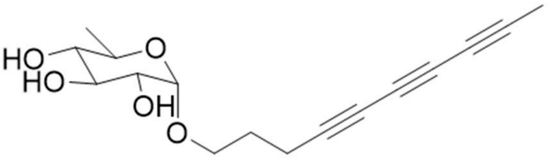

2

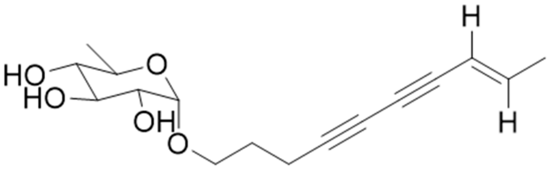

3

Figure S34. Key HMBC (red arrows) and ^{1}H- ^{1}H COSY correlations (blue bold lines) of **1–3**

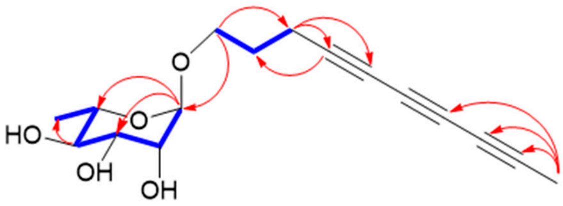

1

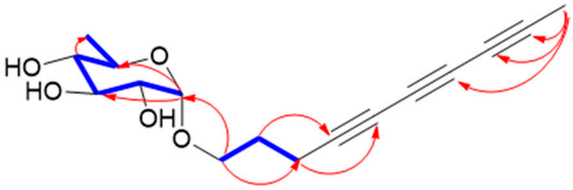

2

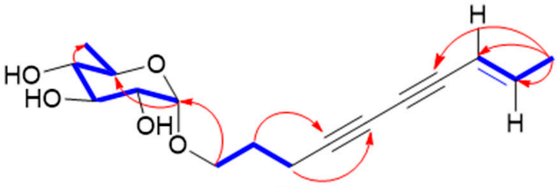

3

Figure S35. Key ROESY correlations (blue double arrows) of **1–3**.

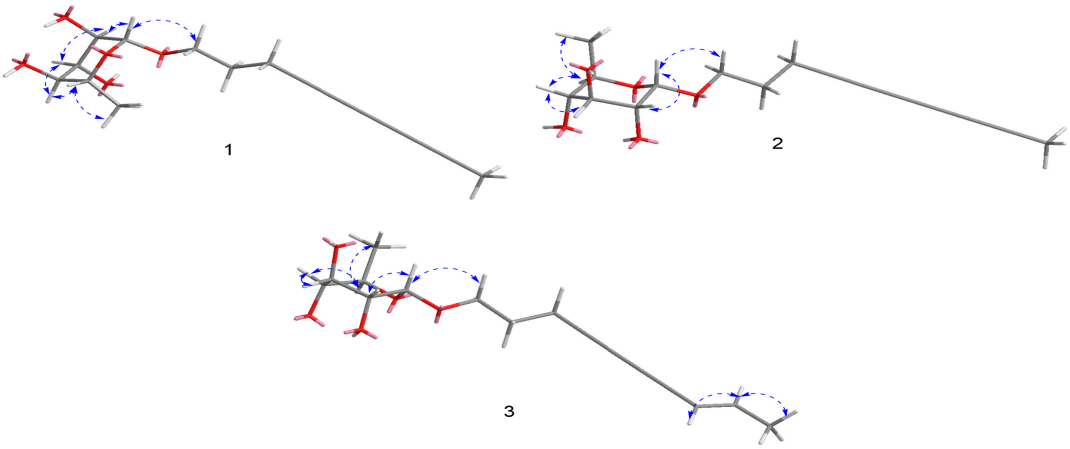

Figure S36. In α -glucosidase inhibitory test, concentration ranges and inhibition rates (I) of acarbose and Compounds 1-3.

Acarbose		Compound 1		Compound 2		Compound 3	
Concentration (μg/mL)	inhibition rate (I)	Concentration (μg/mL)	inhibition rate (I)	concentration (μg/mL)	inhibition rate (I)	Concentration (μg/mL)	inhibition rate (I)
10.00	99.85 %	125.00	93.0 %	125.00	93.10 %	250.00	67.60 %
2.00	99.03 %	62.50	71.00 %	62.50	83.10 %	125.00	58.90 %
1.50	87.90 %	31.25	48.00 %	31.25	76.60 %	62.50	53.90 %
0.78	79.59 %	15.63	29.70 %	15.63	60.40 %	31.25	39.40 %
0.04	64.59 %	7.81	21.90 %	7.81	54.60 %	15.63	24.70 %
0.02	38.07 %	3.91	14.3 %	3.91	32.00 %	7.81	16.70 %
0.01	23.89 %					3.91	3.60 %

Figure S37. Fungal inhibition experiment of compounds **1-3**.

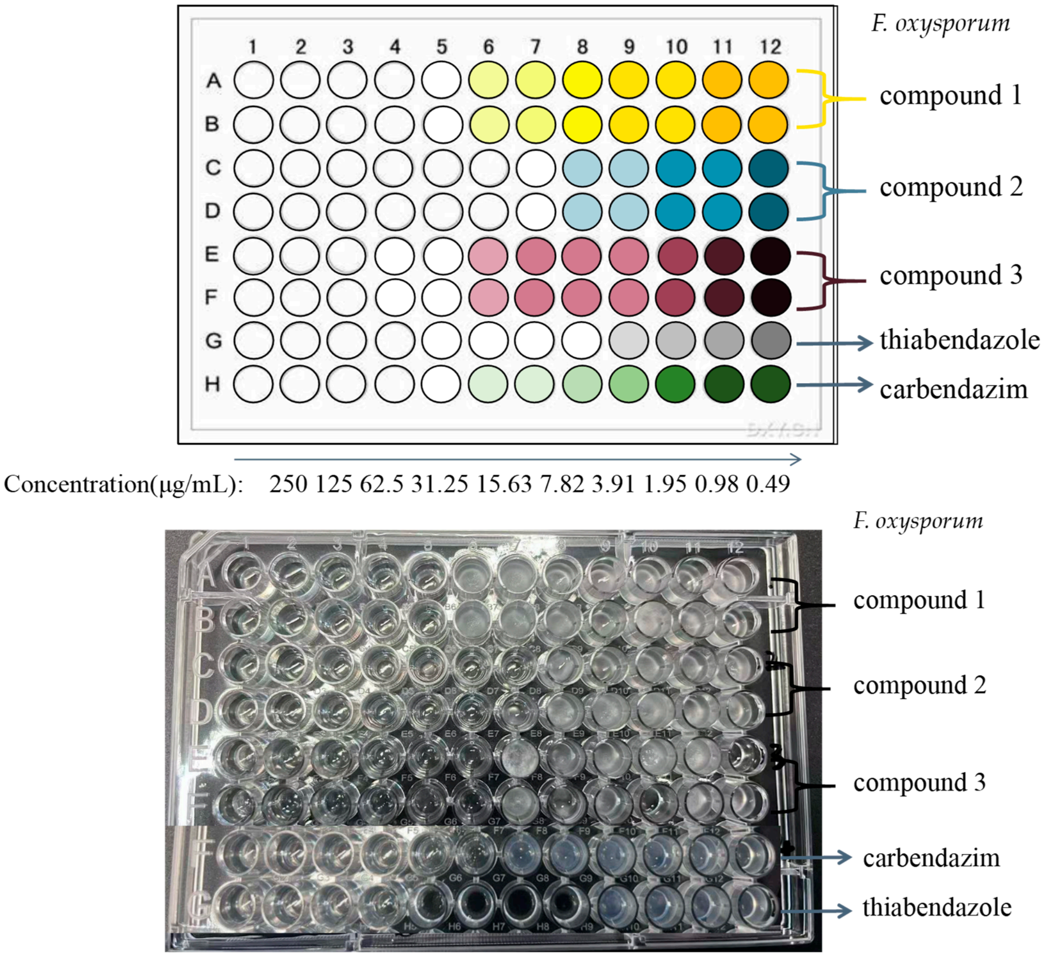

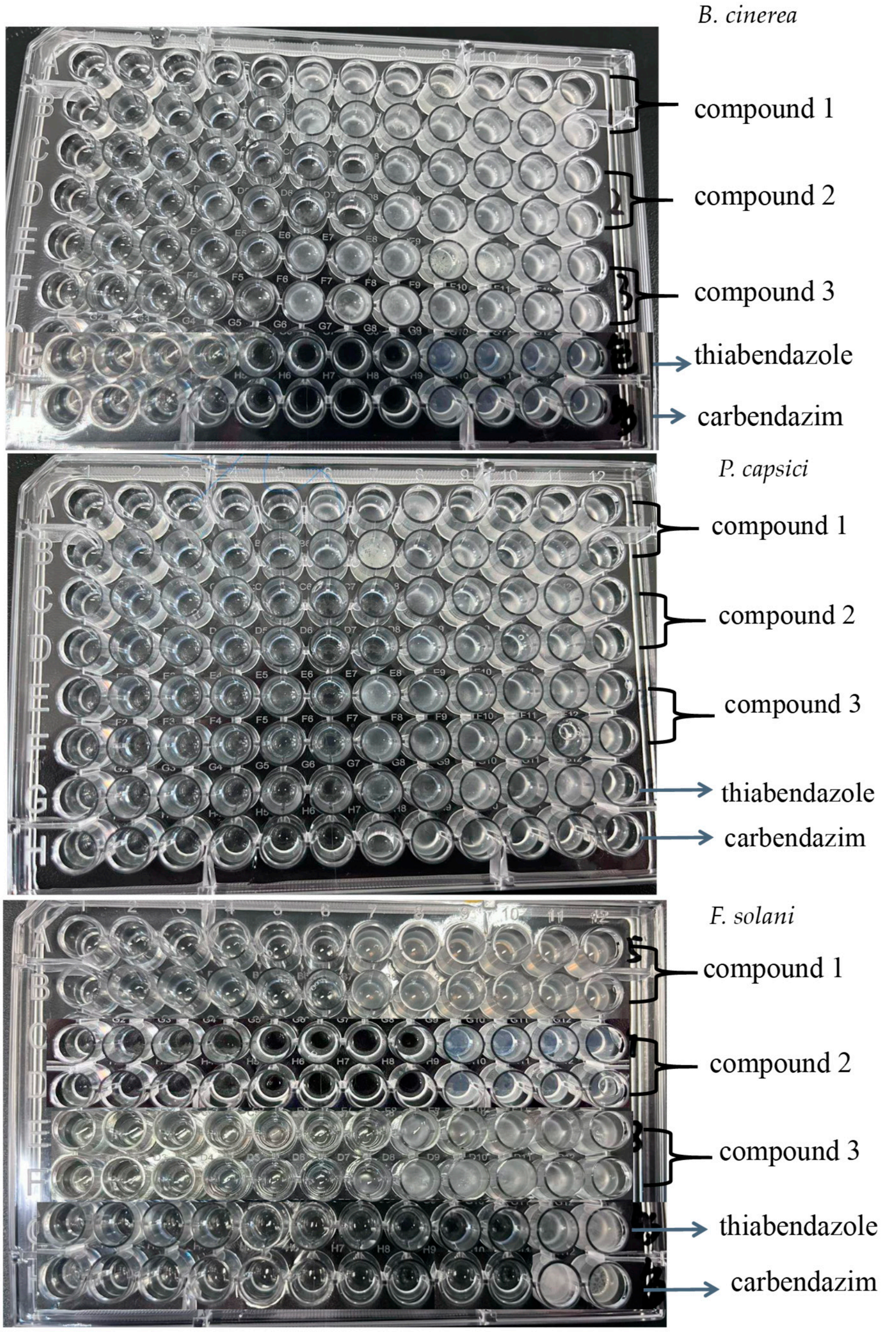
B. cinerea
compound 1
compound 2
compound 3
thiabendazole
carbendazim
P. capsici
compound 1
compound 2
compound 3
thiabendazole
carbendazim
F. solani
compound 1
compound 2
compound 3
thiabendazole
carbendazim

Figure S38. Summary of Experimental Data on *In vivo* Antifungal Activity Assay in Tomatoes

Experimental Group	Independent Experiment Number	Mean Value of Replicate Sub-groups	Mean Value of Three Independent Experiments	Standard Deviation of Three Independent Experiments
	1	1.80		
Compound 1	2	2.00	1.98	0.143372088
	3	2.15		
	1	1.30		
Compound 2	2	1.23	1.14	0.17907168
	3	0.89		
	1	2.32		
Compound 3	2	2.35	2.26	0.111455023
	3	2.10		
	1	4.91		
Blank Control	2	4.70	4.86	0.115758369
	3	4.97		
	1	0.75		
carbendazim	2	0.80	0.74	0.057927157
	3	0.66		
	1	0.74		
thiabendazole	2	0.85	0.82	0.0601849
	3	0.88		

In the table, the mean value of replicate sub-groups represents the average of the data from 3 replicate sub - groups within each group. The mean value of three independent experiments is the average of the mean values of replicate sub-groups from three independent experiments. The standard deviation of three independent experiments reflects the degree of dispersion of the data from three independent experiments.

Figure S39. Summary of Experimental Data on *In vivo* Antifungal Activity Assay in Strawberry

Experimental Group	Independent Experiment Number	Mean Value of Replicate Sub-groups	Mean Value of Three Independent Experiments	Standard Deviation of Three Independent Experiments
	1	1.73		
Compound 1	2	1.74	1.86	0.181536651
	3	2.12		
	1	0.56		
Compound 2	2	0.75	0.68	0.083399973
	3	0.72		
	1	2.04		
Compound 3	2	1.81	1.85	0.141656862
	3	1.70		
	1	4.27		
Blank Control	2	4.41	4.27	0.118415464
	3	4.12		
	1	0.59		
carbendazim	2	0.72	0.66	0.053541261
	3	0,67		
	1	0.50		
thiabendazole	2	0.58	0.60	0.090921211
	3	0.72		

In the table, the mean value of replicate sub-groups represents the average of the data from 3 replicate sub - groups within each group. The mean value of three independent experiments is the average of the mean values of replicate sub-groups from three independent experiments. The standard deviation of three independent experiments reflects the degree of dispersion of the data from three independent experiments.
